# HDAC1 Regulates Acquired Resistance to EGFR Inhibitors through the TFCP2-NDRG1 Signaling Axis in Pancreatic Cancer

**DOI:** 10.7150/ijbs.131003

**Published:** 2026-04-08

**Authors:** Taoyu Chen, Yuxuan Li, Yan Sun, Qixun Fu, Heshui Wu, Dan Li, Dianyun Ren

**Affiliations:** 1 Department of Pancreatic Surgery, Union Hospital, Tongji Medical College, Huazhong University of Science and Technology, Wuhan 430022, Hubei, China.; 2 Sino-German Laboratory of Personalized Medicine for Pancreatic Cancer, Union Hospital, Tongji Medical College, Huazhong University of Science and Technology, Wuhan 430022, Hubei, China.; 3 Department of Critical Care Medicine, Union Hospital, Tongji Medical College, Huazhong University of Science and Technology, Wuhan, Hubei, China.; 4 Department of Thoracic Surgery, Union Hospital, Tongji Medical College, Huazhong University of Science and Technology, Wuhan, Hubei, China.

**Keywords:** histone deacetylase1, transcription factor CP2, PDAC, erlotinib resistance, therapeutic strategy

## Abstract

Epidermal growth factor receptor (EGFR) is a pivotal therapeutic target in pancreatic ductal adenocarcinoma (PDAC); however, the clinical efficacy of tyrosine kinase inhibitors (TKIs) such as erlotinib is frequently curtailed by acquired resistance. This study identifies histone deacetylase 1 (HDAC1) as a critical epigenetic driver of this resistance. HDAC1 is markedly upregulated in erlotinib-resistant PDAC cells, where it directly suppresses the transcriptional activity of TFCP2 through site-specific deacetylation at lysine 256 (K256). This modification attenuates TFCP2 function, leading to transcriptional repression of the metastasis suppressor NDRG1 and increased expression of EGFR, thereby activating EGFR-TKI resistance signaling pathways. Furthermore, EGFR-mediated tyrosine phosphorylation protects HDAC1 from ubiquitin-proteasome system (UPS)-dependent degradation, stabilizing HDAC1 and establishing a self-reinforcing feedback loop that sustains its elevated expression in the resistant state. To counter this mechanism, we designed a bioactive peptide derived from TFCP2 that competitively inhibits K256 deacetylation, thereby restoring TFCP2 transcriptional activity. In *vitro* and in *vivo* studies demonstrate that pharmacological inhibition of HDAC1 or restoration of TFCP2 acetylation reverses erlotinib resistance in PDAC. These findings unveil a previously unrecognized mechanism of EGFR-TKI resistance and suggest a promising strategy to enhance therapeutic efficacy in PDAC.

## Introduction

Pancreatic cancer (PC), particularly pancreatic ductal adenocarcinoma (PDAC), is one of the most lethal malignancies in the gastrointestinal tract, with a five-year survival rate of less than 10%^1^. Surgical resection remains the only potentially curative treatment; however, nearly 80% of patients are diagnosed at advanced stages, which renders them ineligible for surgery^1^, ^2^. Consequently, chemotherapy with gemcitabine or 5-fluorouracil remains the primary treatment, though its clinical benefit is limited. Advances in genomic profiling have identified key driver mutations, including KRAS, TP53, CDKN2A, and SMAD4, providing a foundation for ongoing targeted therapy efforts^3^. However, approved targeted therapies benefit only a small subset of patients with specific molecular alterations (e.g., BRCA mutations), and their overall efficacy in PDAC is markedly lower than in other solid tumors^4^, ^5^. Urgent breakthroughs are needed to enhance therapeutic outcomes in this field.

The epidermal growth factor receptor (EGFR), also known as HER1 or ErbB1, is a critical member of the receptor tyrosine kinase (RTK) family. As a key regulator of cellular processes, EGFR governs cell proliferation, migration, and differentiation, and is essential for tissue homeostasis, regeneration, and tumorigenesis^6^. EGFR is frequently overexpressed and strongly associated with poor prognosis, local invasion, and distant metastasis in PDAC^7^, ^8^. Therapeutic targeting of EGFR not only suppresses PDAC progression but also enhances the antitumor efficacy of gemcitabine^9^. Anti-EGFR agents inhibit the expression of pro-angiogenic factors, thereby disrupting angiogenesis, impairing tumor invasiveness, and sensitizing tumors to chemotherapy and radiotherapy^10^. Current EGFR-targeted therapies include monoclonal antibodies and small-molecule tyrosine kinase inhibitors (TKIs), the latter of which competitively bind the ATP-binding pocket of EGFR's intracellular kinase domain, preventing receptor autophosphorylation and downstream signal transduction^11^.

Erlotinib, a quinazoline-derived small-molecule EGFR-TKI, reversibly inhibits EGFR kinase activity via ATP-competitive binding^12^. Its antitumor efficacy has been demonstrated across various malignancies, including non-small cell lung cancer (NSCLC), ovarian cancer, and head and neck squamous cell carcinoma^10^. The U.S. Food and Drug Administration (FDA) has approved erlotinib in combination with gemcitabine for the treatment of advanced PDAC^13^. However, monotherapy with EGFR TKIs in PDAC offers only modest clinical benefits, primarily due to the emergence of resistance mechanisms that bypass EGFR blockade^14^. These mechanisms include EGFR gene alterations, activation of alternative signaling pathways, reactivation of downstream signaling, and phenotypic changes such as epithelial-mesenchymal transition (EMT)^15^.

Histone deacetylase 1 (HDAC1), a member of the class I HDAC family, catalyzes the removal of acetyl groups from lysine residues on nuclear histones, promoting chromatin compaction and transcriptional repression^16^. Beyond histones, HDAC1 also regulates non-histone proteins and plays a central role in epigenetic modulation. Aberrant HDAC1 overexpression has been implicated in tumor progression and poor prognosis across multiple solid cancers, including gastric, breast, and colorectal carcinomas^17^, ^18^. In PDAC, HDAC1 contributes to gemcitabine resistance by remodeling chromatin accessibility and modulating resistance-related pathways such as EMT, cell cycle progression, and apoptosis^19^. Moreover, HDAC1 promotes PDAC proliferation and metastasis by regulating mutant p53 activity and repressing CDH1 transcription^20^, ^21^.

In this study, we identify HDAC1 as a critical epigenetic regulator of acquired resistance to erlotinib in PDAC. Mechanistically, HDAC1 mediates the deacetylation of the transcription factor TFCP2 at lysine 256 (K256), thereby diminishing its transcriptional activity. This suppression leads to downregulation of NDRG1 and activation of EGFR-TKI resistance pathways. Furthermore, EGFR-driven tyrosine phosphorylation prevents UPS-mediated degradation of HDAC1, leading to its stabilization and creating a self-sustaining feedback loop that maintains high HDAC1 expression in resistant cells. Importantly, pharmacologic inhibition of HDAC1 or competitive restoration of TFCP2 acetylation effectively reverses the resistant phenotype, providing a strong rationale for HDAC1-targeted strategies to overcome EGFR-TKI resistance in PDAC.

## Results

### HDAC1 is upregulated in PDAC with acquired resistance to erlotinib

**Figure 1:**
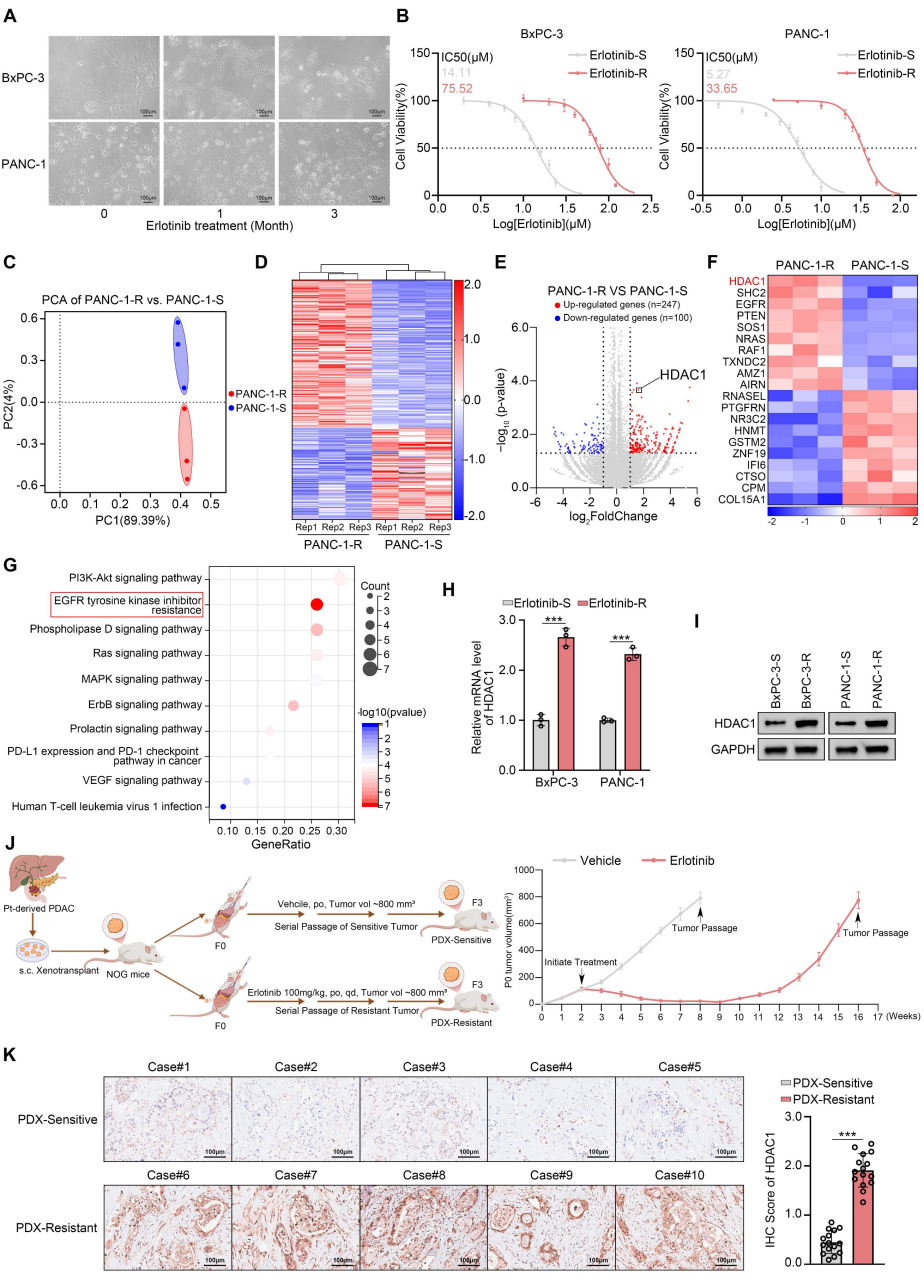
** HDAC1 is upregulated in PDAC with acquired resistance to erlotinib. (A)** Parental BxPC-3 and PANC-1 cells were cultured in gradually increasing concentrations of erlotinib for three months. Phase-contrast images were taken at monthly intervals to document morphological changes associated with the development of drug resistance. **(B)** The IC_50_ values for erlotinib were determined in parental and erlotinib-resistant BxPC-3 and PANC-1 cells using the CCK8 assay. **(C)** PCA of RNA-seq data comparing PANC-1-R and PANC-1-S (n = 3). **(D** and** E)** Heatmap and volcano plot show the differentially expressed genes in PANC-1-R and PANC-1-S cells. **(F)** The heatmap depicts the expression levels of a panel of DEGs. **(G)** KEGG pathway enrichment analysis was performed on the 247 upregulated genes. **(H** and** I)** qRT-PCR analysis and Western blot analysis were performed to assess HDAC1 mRNA and protein expression levels in BxPC-3 and PANC-1 cells. Data are mean ± SD. n=3, ***P < 0.001. **(J)** Schematic of the acquired erlotinib-resistant PDX model. **(K)** Representative IHC images and quantification of HDAC1 staining in erlotinib-sensitive and resistant PDX tumor sections. Data are mean ± SD (n = 5 biologically independent repeats and 3 independent IHC quantifications. ns, not significant; ***P < 0.001.

Although erlotinib is approved for the treatment of locally advanced or metastatic PDAC, the emergence of acquired resistance remains a significant clinical challenge^14^. To model this resistance, we established drug-resistant derivatives of the erlotinib-sensitive PDAC cell lines (Fig. 1A-B). To elucidate the molecular mechanisms underlying acquired resistance, we performed transcriptome sequencing on both resistant and parental PANC-1 cells. Differential expression analysis identified 247 upregulated and 100 downregulated genes, including HDAC1 in the resistant cells (Fig. 1C-F). Kyoto Encyclopedia of Genes and Genomes (KEGG) pathway enrichment analysis revealed a significant activation of the EGFR-TKI resistance signaling pathway in PANC-1-R cells (Fig. 1G). Previous studies have demonstrated that HDACis can enhance the sensitivity of various cancers to EGFR-TKIs, but the underlying mechanisms remain unclear^22^-^24^. Among the 247 upregulated genes, HDAC1 was notably elevated, prompting us to investigate its role in mediating erlotinib resistance (Fig. 1E-F). The qRT-PCR and Western blot assays confirmed that HDAC1 expression levels were markedly increased in the erlotinib-resistant cells (Fig. 1H-I). To determine whether this upregulation was isoform-specific, we further examined other Class I HDACs. However, the expression levels of HDAC2 and HDAC3 remained unchanged at both the transcript and protein levels, suggesting a specific role for HDAC1 in the resistant phenotype (Supplementary Fig. S1A-B). To investigate HDAC1 expression in an in *vivo* setting of acquired resistance, we established subcutaneous PDX models in NOG mice using patient-derived pancreatic cancer tissues. The models were then treated with erlotinib while tumor volumes were monitored. Upon the development of resistance, the models were serially passaged to establish stable erlotinib-resistant PDX lines (Fig. 1J). Subsequent immunohistochemical (IHC) analysis revealed that HDAC1 expression was significantly upregulated in the erlotinib-resistant PDX models compared with their drug-sensitive counterparts (Fig. 1K).

### Co-silencing of HDAC1 reverses erlotinib resistance

**Figure 2:**
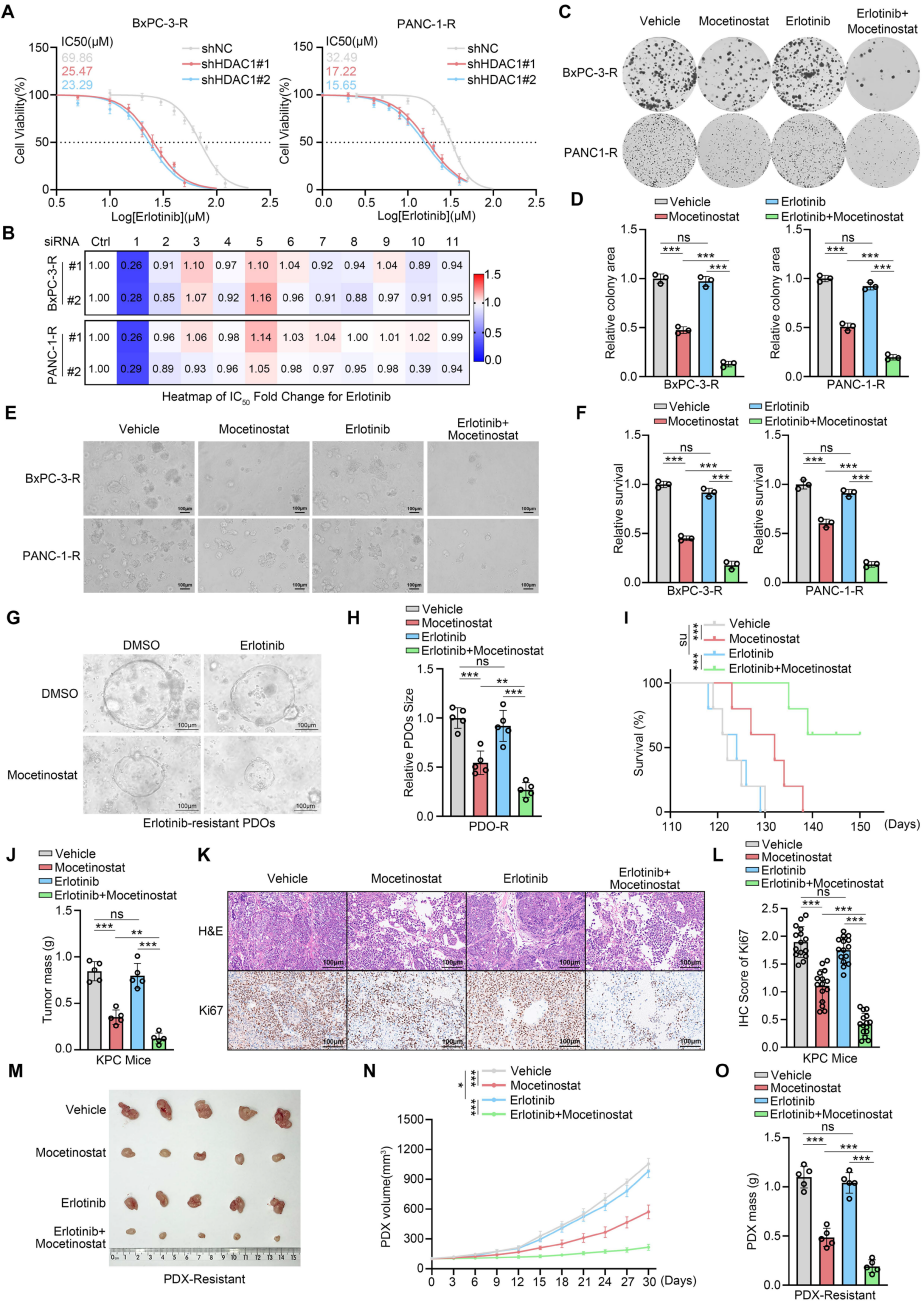
** Co-silencing of HDAC1 reverses erlotinib resistance. (A)** IC_50_ values of erlotinib in HDAC1-knockdown BxPC-3-R and PANC-1-R cells, determined by CCK-8 assay. **(B)** Heatmap showing the fold change in erlotinib IC_50_ after infection with the indicated siRNAs. **(C** and **D)** Colony formation assays in BxPC-3-R and PANC-3-R cells treated with vehicle, mocetinostat (0.15 μM), erlotinib (20 μM), or their combination. Representative images and quantification are shown. Data are mean ± SD (n=3). ***P < 0.001. **(E** and** F)** 3D culture assays of BxPC-3-R and PANC-1-R cells under the same treatment conditions as in **(C)**. Representative images and quantification are shown. Data are mean ± SD (n=3). ***P < 0.001. **(G** and** H)** Representative images and size quantification of PDO-R treated with vehicle, mocetinostat (0.5 μM), erlotinib (50 μM), or their combination. Data are mean ± SD (n=5). ***P < 0.001. **(I)** Survival analysis of KPC mice by Kaplan-Meier curves; n=5, log-rank test. **(J)** Tumor weights in KPC mice. Data are mean ± SD (n=5). ns, not significant, **P < 0.01, ***P < 0.001. **(K** and** L)** Representative IHC images and quantitative IHC scores of tumors from KPC mice. n=5 biologically independent repeats and 3 independent IHC quantifications. Data are mean ± SD. ns, not significant, ***P < 0.001. **(M)** Representative tumor images from PDX-R were collected and photographed following euthanasia on day 30. **(N** and** O)** Tumor volumes at each time point and tumor weights on day 30 in PDX-R models treated with vehicle, mocetinostat (80 mg/kg, p.o., q.d.), erlotinib (100 mg/kg, p.o., q.d.), or their combination. Tumor volumes were measured every 3 days. Tumor weights on day 30. Data are mean ± SD (n=3). ns, not significant, *P < 0.05, ***P < 0.001.

To definitively establish the pivotal role of HDAC1 upregulation in acquired erlotinib resistance, we performed shRNA-mediated knockdown of HDAC1 followed by drug sensitivity assays. Consistently, HDAC1 depletion pronouncedly restored erlotinib sensitivity in both resistant cell models. The IC_50_ values of erlotinib decreased dramatically from 69.86 μM to 25.47μM and 23.29 μM in BxPC-3-R cells and from 32.49 μM to 17.22 μM and 15.65 μM in PANC-1-R cells upon HDAC1 knockdown (Fig. 2A). To investigate the specificity of this regulation, we simultaneously knocked down HDAC1 through HDAC11 and found that only HDAC1 depletion restored erlotinib sensitivity (Fig. 2B). This observation was further supported by treatment with both pan-HDAC and HDAC1-specific inhibitors, while inhibitors targeting other HDAC isoforms had no such effect (Supplementary Fig. S1C). We further evaluated mocetinostat, a class I HDAC inhibitor with high selectivity for HDAC1, which has previously demonstrated potent antitumor effects via induction of apoptosis and autophagy in leukemia, lymphoma, and advanced solid tumors^25^, ^26^. Consistent with genetic suppression, mocetinostat markedly sensitized BxPC-3-R and PANC-1-R cells to erlotinib (Supplementary Fig. S1C). Colony formation assays confirmed that mocetinostat effectively reversed resistance in these cell lines, and its combination with erlotinib strongly suppressed cell proliferation (Fig. 2C-D). These findings were further validated by 3D culture models and CCK-8 assays (Fig. 2E-F and Supplementary Fig. S1D). Additionally, we established patient-derived organoids (PDOs) exhibiting acquired erlotinib resistance (Supplementary Fig. S1E). In this clinically relevant model, co-treatment with mocetinostat also effectively restored erlotinib sensitivity (Fig. 2G-H).

To evaluate the therapeutic efficacy of the mocetinostat-erlotinib combination in *vivo*, we established an erlotinib-resistant model in KPC (LSL- KRAS G12D/+; LSL-Trp53R172H/+; Pdx-1-Cre) mice (Supplementary Fig. S1F). In this model, the combination therapy significantly enhanced the response to erlotinib, reducing tumor burden and markedly prolonging survival (Fig. 2I-J). IHC analysis showed a substantial decrease in Ki67 expression in resistant tumors following combination treatment (Fig. 2K-L). Furthermore, in an erlotinib-resistant PDX model, mocetinostat substantially restored erlotinib sensitivity, and its combination with erlotinib potently inhibited tumor growth (Fig. 2M-O and Supplementary Fig. S1G). In summary, co-silencing of HDAC1 effectively reverses acquired erlotinib resistance in PDAC, with this therapeutic benefit consistently recapitulated across multiple preclinical models.

### HDAC1 promotes erlotinib resistance by downregulating NDRG1

**Figure 3:**
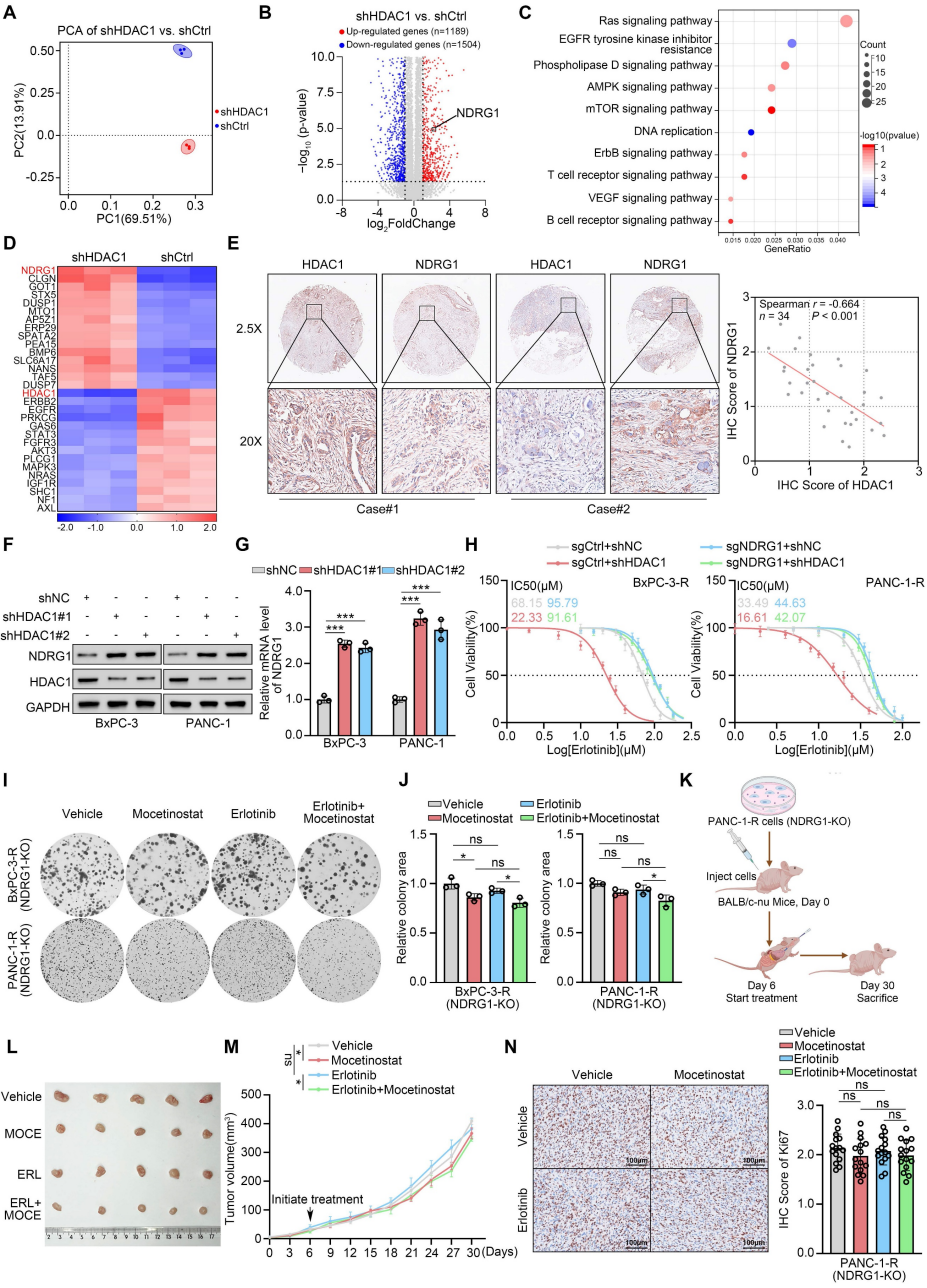
** HDAC1 promotes erlotinib resistance by downregulating NDRG1. (A)** PCA of RNA-seq data comparing shHDAC1 and shCtrl PANC-1 cells (n = 3). **(B)** Volcano plot show the DEGs in PANC-1 cells infected with shHDAC1 or shCtrl. Red dots represent upregulated genes (n=1189), while blue dots represent downregulated genes (n=1504). **(C)** KEGG pathway enrichment analysis was performed on the 1504 downregulated genes. **(D)** The heatmap depicts the expression levels of a panel of DEGs.** (E)** Expression of HDAC1 and NDRG1 was assessed by IHC on a pancreatic cancer TMA. (Left) Representative IHC images. (Right) Scatter plot analysis reveals the correlation between HDAC1 and NDRG1 protein levels, with the P-value indicated.** (F** and** G)** Western blot and qRT-PCR analysis were used to determine the protein expression level and mRNA expression level of NDRG1 in BxPC-3 and PANC-1 cells infected with the indicated shRNAs. Data are mean ± SD (n=3). ***P < 0.001.** (H)** Erlotinib IC_50_ values in NDRG1-knockout BxPC-3-R and PANC-1-R cells, with or without HDAC1 knockdown, assessed by CCK-8 assay. **(I** and **J)** Colony formation assays in NDRG1-knockout BxPC-3-R and PANC-1-R cells treated with vehicle, mocetinostat (0.15 μM), erlotinib (20 μM), or their combination. Representative images (I) and quantification (J) are shown. Data are mean ± SD (n=3). ***P < 0.001. **(K)** Schematic illustration of the in *vivo* subcutaneous tumor model establishment and treatment regimen using NDRG1-knockout PANC-1-R cells in BALB/c-nu mice. **(L)** Representative tumor images from subcutaneous tumor model were collected and photographed following euthanasia on day 30. **(M)** Tumor growth curves of mice treated with mocetinostat (80 mg/kg, p.o., q.d.), erlotinib (100 mg/kg, p.o., q.d.), or their combination. Tumor volumes were measured every 3 days. Data are mean ± SD (n=5). ns, not significant; *P < 0.05. **(N)** IHC analysis of subcutaneous tumors. (Left) Representative images. (Right) Quantification of IHC scores. Scale bars=100μm. n=5 biologically independent repeats and 3 independent IHC quantifications. Data are mean ± SD. ns, not significant.

The above experiments demonstrate that HDAC1 inhibition effectively reverses acquired erlotinib resistance in PDAC. To further elucidate the regulatory mechanisms, we performed RNA sequencing on PANC-1 cells with and without HDAC1 silencing. This analysis identified 1,189 upregulated and 1,504 downregulated genes in HDAC1-silenced cells (Fig. 3A-B and Supplementary Fig. S2A). KEGG pathway enrichment analysis revealed significant downregulation of the EGFR-TKI resistance signalling pathway following HDAC1 knockdown (Fig. 3C). Notably, expression of N-myc downstream-regulated gene 1 (NDRG1) was markedly increased upon HDAC1 silencing (Fig. 3D). Consistently, IHC analysis of a clinical pancreatic cancer tissue microarray (TMA) confirmed a significant negative correlation between HDAC1 and NDRG1 protein levels (Fig. 3E). Western blot and qRT-PCR analyses confirmed a significant increase in NDRG1 expression at both the mRNA and protein levels following HDAC1 silencing (Fig. 3F-G). Treatment with mocetinostat also upregulated both NDRG1 protein and mRNA levels in PC cells (Supplementary Fig. S2B-C). In addition, overexpression of wild-type HDAC1 (WT) suppressed NDRG1 expression, whereas the catalytically inactive HDAC1-D181A mutant had no such effect, indicating that HDAC1-mediated repression of NDRG1 is strictly dependent on its deacetylase activity (Supplementary Fig. S2D-E). This finding is consistent with previous studies demonstrating that HDAC1 functions in a deacetylase activity-dependent manner^27^-^29^.

NDRG1, a key metastasis suppressor, has been shown to inhibit tumor progression by regulating epithelial-mesenchymal transition (EMT), cell migration and angiogenesis^30^. It can also suppress EGFR expression, block its phosphorylation, and reduce EGFR membrane localization via Cav1-mediated endocytosis, thereby enhancing the sensitivity of colorectal cancer cells to the EGFR-targeting monoclonal antibody cetuximab (CTX)^31^. Building on these observations, we further investigated the regulatory mechanism by which NDRG1 suppresses EGFR in PDAC. Western blot analyses showed that NDRG1 knockdown markedly increased total EGFR, p-EGFR, and Cav1 protein levels, suggesting that loss of NDRG1 promotes EGFR accumulation and sustained activation (Supplementary Fig. S2F). Cell-surface biotinylation assays further revealed that NDRG1 depletion accelerated EGFR endocytosis through Cav1 upregulation in pancreatic cancer cells (Supplementary Fig. S2G-H). Unexpectedly, however, co-silencing Cav1 failed to restore erlotinib sensitivity (Supplementary Fig. S2I). These findings indicate that although the NDRG1-Cav1 axis regulates EGFR endocytosis at the molecular level, this process is not the principal driver of erlotinib resistance in pancreatic cancer. This discrepancy may reflect fundamental differences in the mechanisms of action between EGFR-TKIs and EGFR-targeting monoclonal antibodies. Previous studies have shown that NDRG1 directs EGFR to lysosomes for degradation, thereby attenuating EGFR signaling^32^. We thus hypothesized that NDRG1 similarly regulates EGFR protein stability in PDAC. To test this hypothesis, cells were treated with the lysosomal inhibitor Bafilomycin A1 (Baf-A1) or the proteasome inhibitor MG132. These experiments revealed that NDRG1 regulates EGFR predominantly via the lysosomal degradation pathway (Supplementary Fig. S2J-K). Subcellular fractionation and immunoblotting further demonstrated that NDRG1 overexpression markedly elevated EGFR levels in the lysosomal fraction, whereas NDRG1 knockdown reduced them; the opposite trend was observed in other cellular compartments (Supplementary Fig. S2L). Cycloheximide (CHX) chase assays revealed that NDRG1 depletion significantly prolonged EGFR half-life. Notably, lysosomal inhibition by Baf-A1 abolished NDRG1's effect on EGFR stability (Supplementary Fig. S3A-B). Collectively, these findings indicate that while NDRG1 loss promotes EGFR internalization, it predominantly impairs lysosomal degradation post-endocytosis. Consequently, EGFR accumulates intracellularly and remains persistently activated, thereby driving erlotinib resistance in pancreatic cancer cells.

Given the pronounced induction of NDRG1 upon HDAC1 knockdown, we hypothesized that HDAC1 drives erlotinib resistance by repressing NDRG1. To test this, we generated NDRG1-knockout (NDRG1-KO) BxPC-3 and PANC-1 cell lines using CRISPR-Cas9 (Fig. S3C). Drug sensitivity assays showed that HDAC1 knockdown failed to restore erlotinib sensitivity in NDRG1-KO PC cells (Fig. 3H). Similarly, mocetinostat treatment did not enhance erlotinib responsiveness in NDRG1-KO erlotinib-resistant PC cells (Fig. S3D). CCK-8, clonogenic, and 3D cell culture assays further confirmed that mocetinostat was ineffective in overcoming resistance in these cells, and combination treatment with erlotinib did not inhibit cell proliferation (Fig. 3I-J and Supplementary Fig. S3E-G). To evaluate therapeutic efficacy in *vivo*, we established a subcutaneous xenograft model using NDRG1-KO PANC-1-R cells and treated mice with mocetinostat, erlotinib, or both (Fig. 3K). Mocetinostat showed markedly reduced antitumor activity in NDRG1-deficient tumors, and the combination with erlotinib failed to reduce tumor burden or suppress tumor growth (Fig. 3L-M and Supplementary Fig. S3H). IHC analysis revealed that combination treatment did not effectively inhibit Ki67 expression in NDRG1-KO tumors (Fig. 3N). In summary, these results identify NDRG1 as a critical mediator of HDAC1-dependent erlotinib sensitivity in pancreatic cancer, and reveal that HDAC1 promotes erlotinib resistance by repressing NDRG1 expression.

### HDAC1 Regulates NDRG1 Expression by Binding to TFCP2

**Figure 4:**
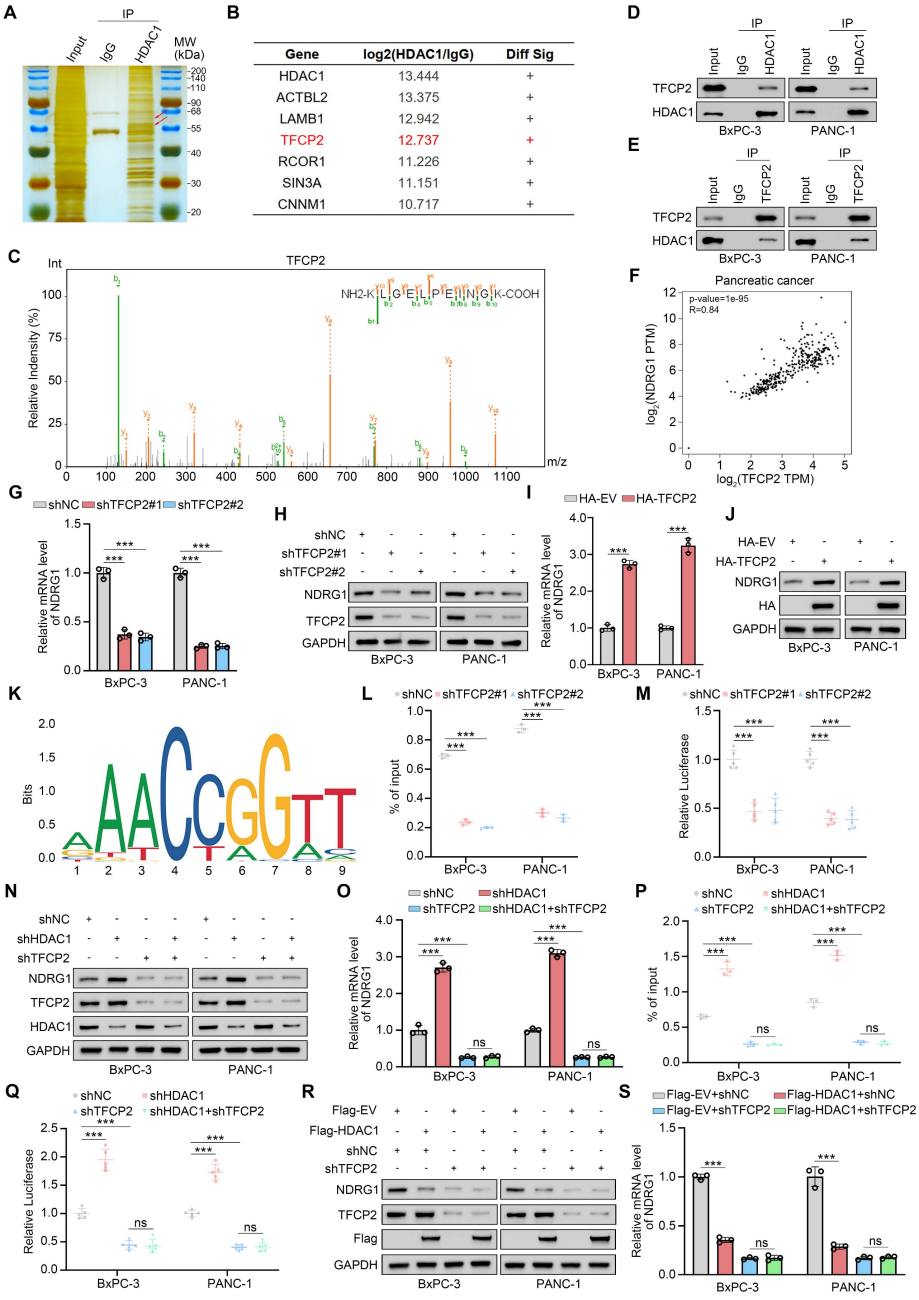
** HDAC1 Regulates NDRG1 Expression by Binding to TFCP2. (A)** Silver staining of HDAC1 immunoprecipitates from PANC-1 cells. The red arrow indicates the predicted molecular weights of TFCP2 and HDAC1. **(B** and** C)** Mass spectrometry of HDAC1 immunocomplexes identifies TFCP2 peptides, indicating a potential HDAC1-TFCP2 interaction. **(D** and** E)** Co-IP confirms the interaction between HDAC1 and TFCP2 in BxPC-3 and PANC-1 cells. **(F)** Correlation between TFCP2 and NDRG1 mRNA expression in PDAC samples analyzed using the GEPIA database. P and R values are shown. **(G** and **H)** qRT-PCR and immunoblot analyses of NDRG1 expression in BxPC-3 and PANC-1 cells transduced with the indicated shRNAs. **(I** and **J)** qRT-PCR and immunoblot analyses of NDRG1 expression in cells expressing HA-TFCP2. **(K)** Predicted TFCP2-binding motifs within the NDRG1 promoter identified by JASPAR database analysis. **(L)** ChIP-qPCR showing TFCP2 enrichment at NDRG1 promoter regions in BxPC-3 and PANC-1 cells expressing the indicated shRNAs (n=3). Data are mean ± SD; ***P < 0.001. **(M)** Luciferase reporter assays assessing NDRG1 promoter activity in BxPC-3 and PANC-1 cells expressing the indicated shRNAs (n=5). Data are mean ± SD; ***P < 0.001. **(N-Q)** Effects of HDAC1 and TFCP2 knockdown on NDRG1 regulation. **(N-O)** Protein and mRNA levels of NDRG1 analyzed by immunoblotting and qRT-PCR. **(P)** TFCP2 enrichment at NDRG1 promoter analyzed by ChIP-qPCR. **(Q)** Luciferase reporter assays measuring NDRG1 promoter activity. Luciferase, n=5; others, n=3. Data are mean ± SD; ***P < 0.001. **(R** and** S)** NDRG1 protein **(R)** and mRNA **(S)** levels in BxPC-3 and PANC-1 cells infected with the indicated shRNAs.

To dissect the mechanism by which HDAC1 regulates NDRG1, we performed silver staining gel electrophoresis on HDAC1 immunoprecipitates from PANC-1 cells to identify potential interacting proteins based on molecular weight (Fig. 4A). We next performed immunoprecipitation coupled with tandem mass spectrometry (IP-MS) to systematically define HDAC1-binding partners. This analysis revealed several HDAC1 interactors, including ACTBL2, LAMB1, and the transcription factor TFCP2 (Fig. 4B). Given HDAC1's transcriptional regulation of NDRG1, we hypothesized that it modulates NDRG1 transcription via specific transcription factor interactions. Guided by the IP-MS results, we focused on the HDAC1-TFCP2 interaction (Fig. 4C). TFCP2 has been reported to regulate downstream gene expression and drive tumorigenesis^33^. Co-IP confirmed the endogenous interaction between HDAC1 and TFCP2 in pancreatic cancer cells (Fig. 4D-E). Analysis of the GEPIA database revealed a robust positive correlation between TFCP2 and NDRG1 mRNA levels in PDAC (Fig. 4F), Functionally, TFCP2 knockdown markedly decreased NDRG1 mRNA and protein levels, whereas its overexpression induced a corresponding increase (Fig. 4G-J). Bioinformatic analysis of the NDRG1 promoter using JASPAR predicted putative TFCP2 binding motifs (Fig. 4K). ChIP-qPCR and dual-luciferase reporter assays confirmed TFCP2 binding to the NDRG1 promoter and its activation of transcription (Fig. 4L-M and Supplementary Fig. S4A-B). Critically, in TFCP2-depleted cells, HDAC1 inhibition or overexpression failed to alter NDRG1 expression or promoter activity (Fig. 4N-S and Supplementary Fig. S4C-D). In summary, these findings indicate that HDAC1 represses NDRG1 transcription and expression via its interaction with TFCP2.

### HDAC1 deacetylates TFCP2 at K256 to inhibit transcriptional activity

**Figure 5:**
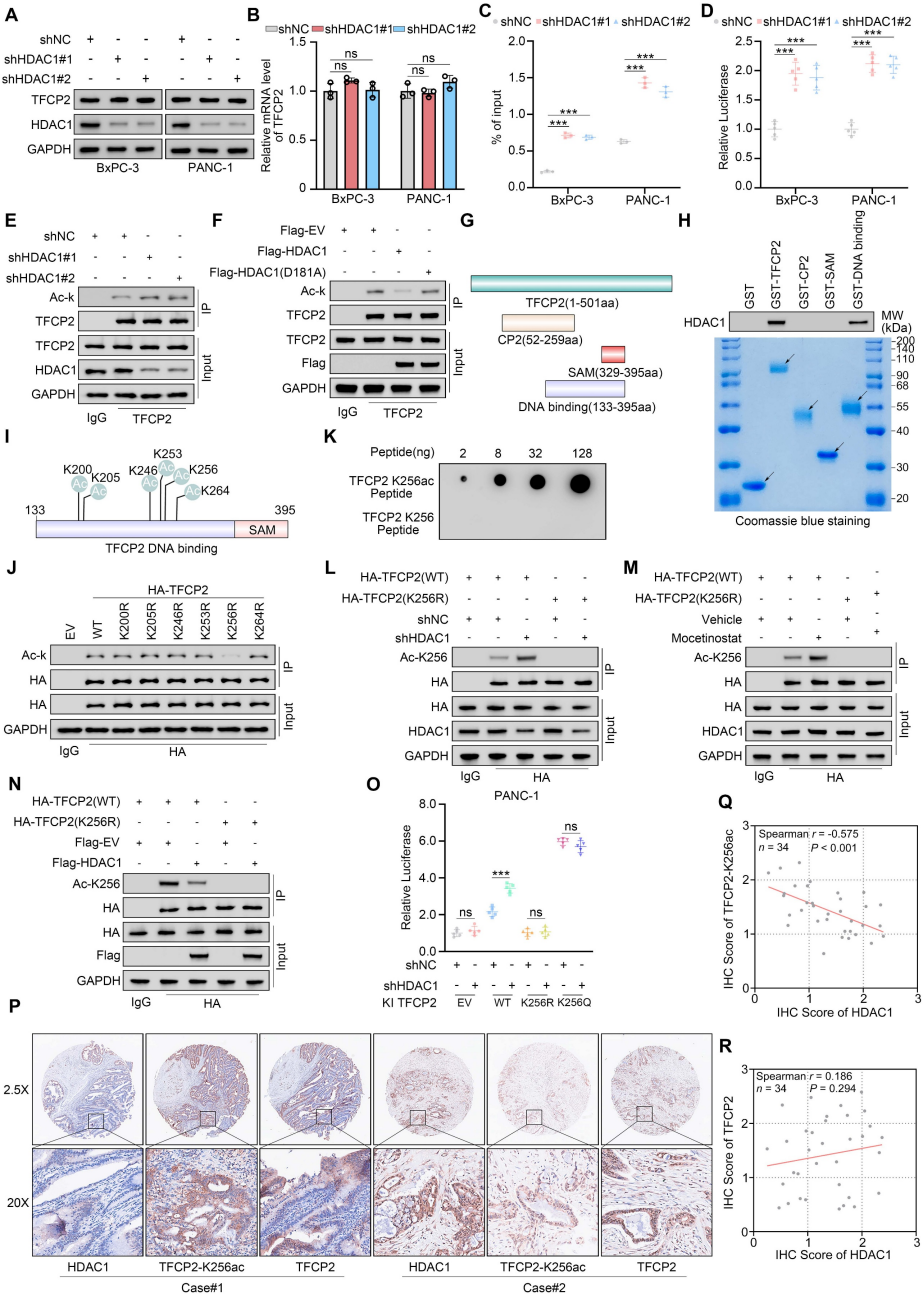
** HDAC1 deacetylates TFCP2 at K256 to inhibit transcriptional activity. (A** and** B)** Western blot (A) and qRT-PCR (B) analyses of TFCP2 protein and mRNA levels in BxPC-3 and PANC-1 cells expressing the indicated shRNAs (n = 3). Data are mean ± SD; ns, not significant. **(C)** ChIP-qPCR analysis of TFCP2 enrichment at the NDRG1 promoter in BxPC-3 and PANC-1 cells expressing the indicated shRNAs (n = 3). Data are mean ± SD; ***P < 0.001. **(D)** Luciferase reporter assays assessing TFCP2 transcriptional activity in cells expressing the indicated shRNAs (n = 5). Data are mean ± SD; ***P < 0.001. **(E** and** F)** Co-IP detecting TFCP2 lysine acetylation in HDAC1-deficient (E) or plasmid-transfected (F) PANC-1 cells. **(G)** Schematic diagrams of the truncations of TFCP2. **(H)** Western blot analysis of HDAC1 pulled down from PANC-1 lysates by GST or GST-TFCP2 fusion proteins. Arrows indicate expected molecular weights. **(I)** Predicted lysine acetylation sites within the DNA-binding domain of TFCP2 based on the CPLM database. **(J)** Co-IP detecting lysine acetylation of exogenous WT or mutant TFCP2 in PANC-1 cells. **(K)** Peptide validation of anti-TFCP2-K256ac antibody by dot blot using synthesized peptides. **(L)** Western blot detection of TFCP2-K256 acetylation in PANC-1 cells transfected with TFCP2 (WT or K256R) and expressing the indicated shRNAs. **(M)** Western blot analysis of TFCP2-K256 acetylation in PANC-1 cells transfected with TFCP2 (WT or K256R) and treated with vehicle or mocetinostat. **(N)** Western blot analysis of TFCP2-K256 acetylation in PANC-1 cells transfected with the indicated plasmids. **(O)** Luciferase reporter activities of TFCP2 were assessed in PANC-1 cells infected with shHDAC1 and knock-in TFCP2 (WT/K256R/K256Q). Data are mean ± SD (n = 5); ns, not significant, ***P < 0.001. **(P-R)** IHC analysis of HDAC1, TFCP2, and TFCP2-K256ac expression in a pancreatic cancer TMA. **(P)** Representative IHC images. **(Q and R)** Scatter plots showing correlations between HDAC1 and TFCP2-K256ac **(Q)**, and between HDAC1 and TFCP2 **(R)**.

To investigate the specific role of HDAC1 in modulating TFCP2 function, we knocked down HDAC1 in BxPC-3 and PANC-1 cells. Western blot and qRT-PCR analyses showed no significant changes in TFCP2 protein or mRNA levels (Fig. 5A-B). In contrast, dual-luciferase reporter assays and ChIP-qPCR revealed that both HDAC1 silencing and HDAC1 inhibitor treatment significantly increased TFCP2 transcriptional activity (Fig. 5C-D and Supplementary Fig. S5A-B). Moreover, neither HDAC1-WT nor HDAC1-D181A affected TFCP2 expression, but HDAC1-WT markedly reduced TFCP2 transcriptional activity (Supplementary Fig. S5C-F). As a histone deacetylase, HDAC1 primarily suppresses transcriptional activity by removing acetyl groups from histones^34^. We hypothesized that HDAC1 modulates TFCP2 activity by regulating its acetylation status. Indeed, HDAC1 knockdown or mocetinostat treatment significantly increased TFCP2 acetylation in PANC-1 cells (Fig. 5E and Supplementary Fig. S5G), while overexpression of HDAC1-WT reduced TFCP2 acetylation, with no effect observed for HDAC1-D181A (Fig. 5F). Overexpression of HDAC2 to HDAC10 did not alter TFCP2 acetylation, further supporting the specificity of HDAC1 (Supplementary Fig. S5H-P).

To identify the HDAC1-interacting acetylation domain in TFCP2, we performed GST pull-down assays using recombinant TFCP2 protein (Fig. 5G). These assays revealed that HDAC1 specifically binds to the DNA-binding domain of TFCP2 (Fig. 5H). Acetylation site prediction using the CPLM database identified six putative lysine residues within this region (Fig. 5I). To validate the key site, we generated acetylation-deficient mutants and expressed HA-tagged TFCP2 (WT and mutants) in TFCP2-depleted (shTFCP2) PANC-1 cells. Co-IP analysis revealed that only the K256R mutant exhibited a significant reduction in acetylation, identifying K256 as the major acetylation site (Fig. 5J). A custom antibody was then generated to detect acetylation at K256 (Fig. 5K and Supplementary Fig. S5Q). Subsequent Co-IP confirmed that HDAC1 knockdown or mocetinostat treatment markedly increased K256 acetylation in TFCP2(WT), whereas HDAC1 overexpression reduced it. Importantly, acetylation of the K256R mutant was not recognized by the antibody, validating its specificity (Fig. 5L-N). Finally, dual-luciferase assays demonstrated that HDAC1 knockdown did not affect the transcriptional activity of either the acetylation-deficient TFCP2(K256R) or the acetylation-mimic TFCP2(K256Q) (Fig. 5O). Notably, TFCP2(K256Q) exhibited significantly enhanced activity compared to TFCP2(WT), while TFCP2(K256R) showed markedly reduced activity (Fig. 5O). Moreover, IHC analysis of a pancreatic cancer TMA revealed that HDAC1 expression was inversely correlated with TFCP2 acetylation at K256 but did not correlate with total TFCP2 expression (Fig. 5P-R). Notably, high HDAC1 expression and diminished TFCP2 K256 acetylation are associated with poor patient prognosis (Supplementary Table S3). In summary, these findings demonstrate that HDAC1 represses TFCP2 transcriptional activity through site-specific deacetylation at K256.

### TFCP2 acetylation sustains the HDAC1-TFCP2-NDRG1-EGFR positive feedback loop

**Figure 6:**
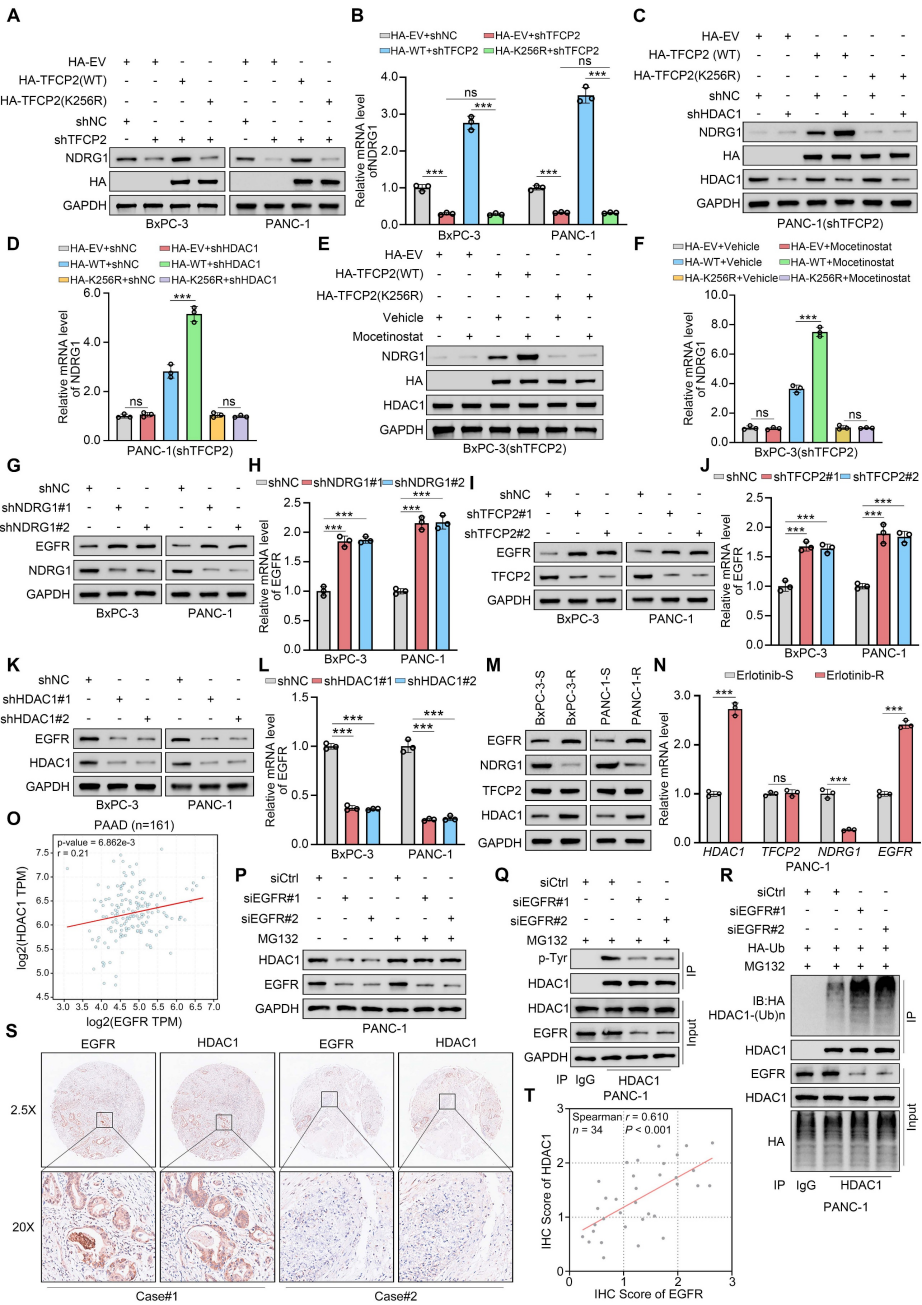
** TFCP2 acetylation sustains the HDAC1-TFCP2-NDRG1-EGFR positive feedback loop. (A** and** B)** Western blot (A) and qRT-PCR (B) showing protein and mRNA levels of NDRG1 in BxPC-3 and PANC-1 cells expressing TFCP2-WT or K256R and transduced with shNC or shTFCP2 (n = 3). Data are mean ± SD; ns, not significant; ***P < 0.001.** (C** and** D)** Western blot (C) and qRT-PCR (D) showing NDRG1 expression in cells expressing TFCP2-WT or K256R and transduced with shNC or shHDAC1 (n = 3). Data are mean ± SD; ns, not significant; ***P < 0.001.** (E** and** F)** Western blot (E) and qRT-PCR (F) showing NDRG1 expression in cells expressing TFCP2-WT or K256R treated with vehicle or mocetinostat (0.15 μM) (n = 3). Data are mean ± SD; ns, not significant; ***P < 0.001. **(G-L)** Western blot and qRT-PCR analyses of EGFR protein and mRNA levels in BxPC-3 and PANC-1 cells transduced with the indicated shRNAs (n = 3). Data are mean ± SD; ***P < 0.001.** (M)** Western blot showing expression of indicated proteins in erlotinib-sensitive and erlotinib -resistant BxPC-3 and PANC-1 cells. **(N)** qRT-PCR analysis of indicated mRNAs in erlotinib-sensitive and -resistant PANC-1 cells (n = 3). Data are mean ± SD; ns, not significant; ***P < 0.001.** (O)** Correlation between TFCP2 and NDRG1 mRNA levels in PDAC samples using CPTAC. P and R values are indicated.** (P)** Western blot of HDAC1 in PANC-1 cells transfected with siRNAs and treated with DMSO or MG132 (10 μM, 8 h). **(Q)** Co-IP assessing tyrosine phosphorylation of HDAC1 in EGFR-deficient PANC-1 cells.** (R)** Co-IP analysis of HDAC1 ubiquitination in EGFR-deficient PANC-1 cells.** (S** and** T)** IHC analysis of EGFR and HDAC1 in a pancreatic cancerTMA. (S) Representative images. (T) Scatter plots showing correlation between EGFR and HDAC1; P values are indicated.

To further investigate the regulatory role of TFCP2 acetylation in controlling NDRG1 expression, we reintroduced either TFCP2 (WT) or TFCP2 (K256R) into TFCP2-depleted BxPC-3 and PANC-1 cells. Reconstitution with TFCP2 (WT) markedly elevated both NDRG1 protein and mRNA levels, whereas the K256R mutant failed to produce a comparable effect (Fig. 6A-B). Upon HDAC1 knockdown, NDRG1 expression was significantly upregulated only in cells reconstituted with TFCP2 (WT), but not in those expressing TFCP2 (K256R), in which NDRG1 levels remained similar to those observed in TFCP2-deficient cells (Fig. 6C-D). Consistent findings were obtained following treatment with mocetinostat in BxPC-3 cells (Fig. 6E-F).

Our previous study showed that HDAC1 suppresses TFCP2 transcriptional activity via deacetylation, thereby downregulating NDRG1 expression and promoting erlotinib resistance in PDAC. Independent studies have reported that NDRG1 enhances sensitivity to EGFR-TKIs in colorectal cancer by downregulating EGFR expression^31^. Based on these findings, we hypothesized that the HDAC1-TFCP2-NDRG1 axis modulates erlotinib sensitivity in PDAC through regulation of EGFR expression. KEGG pathway analysis of our previously generated transcriptomic datasets revealed significant enrichment of the EGFR-TKI resistance pathway—including upregulation of EGFR itself—in erlotinib-resistant PANC-1 cells (Fig. 1F-G). In contrast, HDAC1-depleted PANC-1 cells showed downregulation of this pathway, accompanied by reduced EGFR expression (Fig. 3C-D). Western blotting and qRT-PCR analyses further demonstrated that silencing NDRG1 or TFCP2 markedly increased EGFR protein and mRNA levels, whereas HDAC1 knockdown significantly decreased EGFR expression (Fig. 6G-L). These results were recapitulated upon overexpression of the respective constructs (Supplementary Fig. S6A-F). This regulatory axis was further validated in both BxPC-3 and PANC-1 cells. In resistant cells, HDAC1 expression was significantly elevated, TFCP2 expression remained unchanged, NDRG1 was downregulated, and EGFR was upregulated (Fig. 6M-N and Supplementary Fig. S6G). The functional relevance of this regulatory axis was further confirmed in *vivo* by IHC staining of PDX models (Supplementary Fig. S6H-I).

EGFR activity has been shown to promote HDAC1 tyrosine phosphorylation, enhancing its protein stability^29^. To elucidate the mechanism driving HDAC1 upregulation in erlotinib-resistant cells, we analyzed the CPTAC database, revealing a significant positive correlation between EGFR and HDAC1 mRNA levels (Fig. 6O). Consistent with this, EGFR knockdown markedly reduced both mRNA and protein levels of HDAC1 (Fig. 6P and Supplementary Fig. S6J). Given the role of the UPS in HDAC1 degradation, we found that proteasome inhibition with MG132 effectively rescued the HDAC1 downregulation induced by EGFR silencing (Fig. 6P and Supplementary Fig. S6K). Furthermore, EGFR depletion markedly reduced HDAC1 tyrosine phosphorylation while concurrently augmenting its ubiquitination (Fig. 6Q-R and Supplementary Fig. S6L-M). To establish a causal link between EGFR-mediated phosphorylation and ubiquitin-dependent degradation of HDAC1, we focused on the previously reported Tyr72 phosphorylation site^29^. We generated wild-type (WT) HDAC1 and a phosphorylation-deficient Y72F mutant. Western blot analysis showed that EGFR knockdown markedly reduced the protein level of HDAC1 WT, the Y72F mutant remained largely refractory to EGFR depletion. Notably, the basal protein level of the Y72F mutant was substantially lower than that of the WT protein (Supplementary Fig. S6N). Co-IP assays further showed that, in WT-expressing cells, EGFR depletion significantly decreased HDAC1 tyrosine phosphorylation while concomitantly increasing its ubiquitination. In contrast, EGFR knockdown had no appreciable effect on the phosphorylation or ubiquitination status of the Y72F mutant (Supplementary Fig. S6O-P). IHC staining of a pancreatic cancer TMA further confirmed a significant positive correlation between EGFR and HDAC1 expression (Fig. 6S-T). Collectively, these results indicate that EGFR-mediated tyrosine phosphorylation stabilizes HDAC1 by restricting its UPS-dependent degradation, forming a positive feedback loop (HDAC1-TFCP2-NDRG1-EGFR) that sustains elevated HDAC1 expression in resistant cells.

### Acetylation of TFCP2 at K256 reverses erlotinib resistance

**Figure 7:**
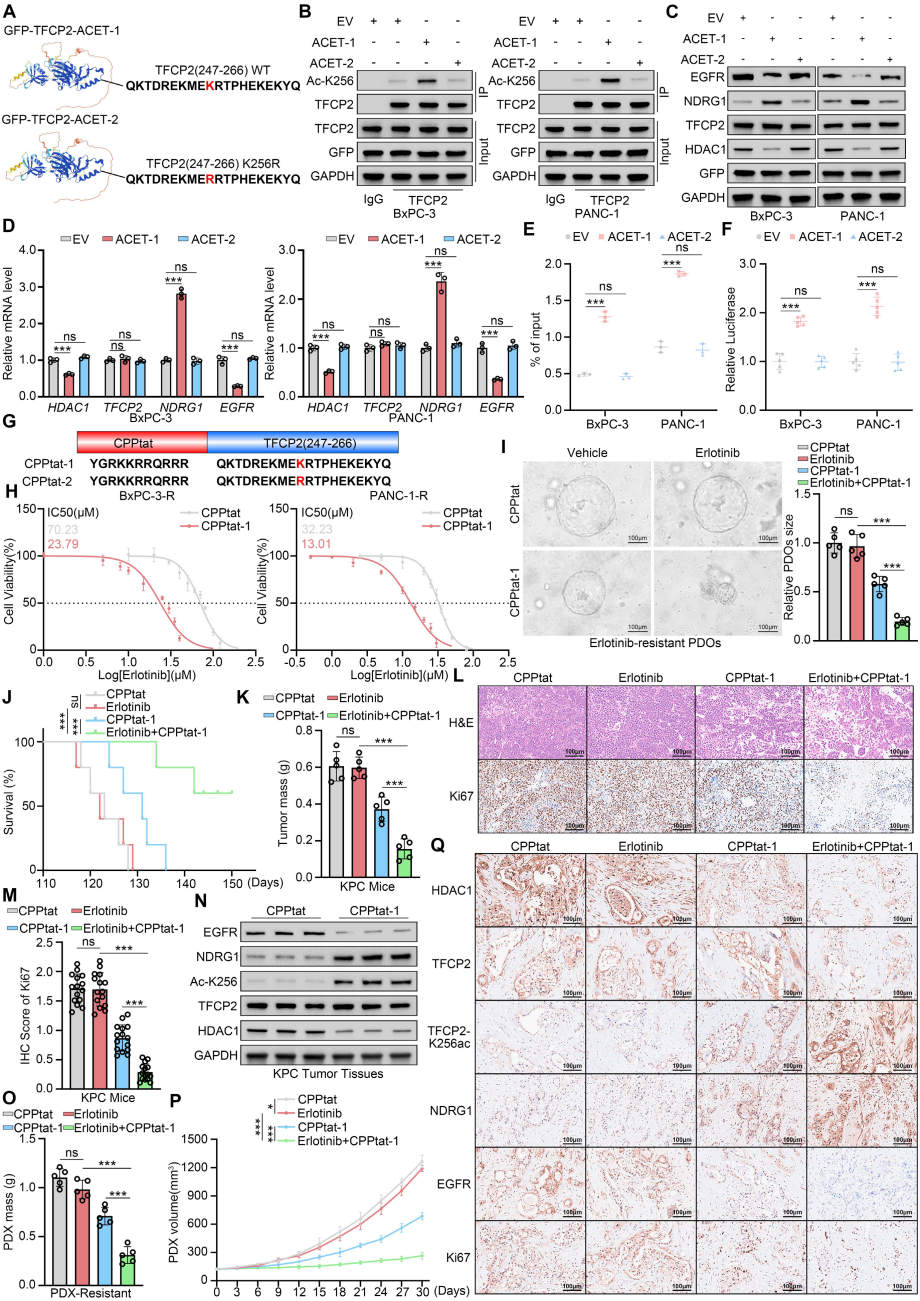
** Acetylation of TFCP2 at K256 reverses erlotinib resistance. (A)** Schematic of GFP fused to TFCP2 (247-266) containing either wild-type or K256R mutant. **(B)** Co-IP showing acetylation at TFCP2 K256 in BxPC-3 and PANC-1 cells transfected with constructs shown in (A). **(C)** Western blot analysis was used to determine the protein expression level of indicated genes in BxPC-3 and PANC-1 cells transfected with the indicated plasmids. **(D)** Western blot (C) and qRT-PCR (D) of indicated proteins and mRNAs in BxPC-3 and PANC-1 cells transfected with constructs shown in (A) (n = 3). Data are mean ± SD; ns, not significant; ***P < 0.001. **(E)** ChIP-qPCR showing TFCP2 enrichment at NDRG1 promoter in cells transfected with indicated plasmids (n = 3). Data are mean ± SD; ns, not significant; ***P < 0.001. **(F)** Luciferase reporter assays assessing TFCP2 transcriptional activity in BxPC-3 and PANC-1 cells (n = 5). Data are mean ± SD; ns, not significant; ***P < 0.001. **(G)** Schematic of cell-penetrating peptides (CPP) fused to wild-type (CPPtat-1) or K256R mutant (CPPtat-2) TFCP2 (247-266). **(H)** IC_50_ values of erlotinib in BxPC-3-R and PANC-1-R cells treated with CPPtat or CPPtat-1 (5 μM) determined by CCK8 assay. **(I)** Representative images and size quantification of PDO-R treated with vehicle, CPPtat-1 (5 μM), erlotinib (50 μM), or their combination (n = 5). Data are mean ± SD; ns, not significant; ***P < 0.001. **(J)** Kaplan-Meier survival analysis of KPC mice (n = 5), analyzed by log-rank test. ns, not significant; ***P < 0.001. **(K)** Tumor weights in KPC mice (n = 5). Data are mean ± SD; ns, not significant; ***P < 0.001. **(L** and **M)** Representative IHC images of tumors (L) and quantification of IHC scores (M). n = 5 biologically independent samples; 3 independent IHC quantifications. Data are mean ± SD; ns, not significant; ***P < 0.001. **(N)** Western blot of indicated proteins in pancreatic tumors from KPC mice treated with CPPtat or CPPtat-1.** (O)** Tumor weights in PDX-R models (n = 5). Data are mean ± SD; ns, not significant; ***P < 0.001. **(P)** Tumor growth curves of PDX-R models treated with CPPtat, CPPtat-1, erlotinib, or combinations. Tumor volumes measured every 3 days (n = 5). Data are mean ± SD; ns, not significant; *P < 0.05; ***P < 0.001. **(Q)** Representative IHC images of PDX-R tumors. Scale bars=100μm.

Our previous study demonstrated that HDAC1-mediated deacetylation of TFCP2 at lysine 256 (K256) contributes to erlotinib resistance in PDAC, suggesting that restoring acetylation at this site may resensitize PDAC cells to erlotinib. Based on the acetylation motif of TFCP2, we designed a peptide corresponding to amino acids 247-266 containing the K256 site (TFCP2-ACET-1) and a K256R mutant (TFCP2-ACET-2), both fused to GFP to competitively inhibit endogenous TFCP2 deacetylation (Fig. 7A). Co-IP assays confirmed that ACET-1 specifically competed with endogenous TFCP2 for K256 deacetylation, markedly restoring acetylation at this site, whereas ACET-2 and the empty vector (EV) control had no such effect (Fig. 7B). Western blot and qRT-PCR analyses revealed that ACET-1 did not affect TFCP2 expression but significantly upregulated NDRG1 at both protein and mRNA levels, concurrently reducing EGFR expression and indirectly inhibiting HDAC1(Fig. 7C-D). ChIP-qPCR and dual-luciferase reporter assays further demonstrated that ACET-1 robustly enhanced TFCP2 transcriptional activity, whereas ACET-2 and EV had no discernible effect (Fig. 7E-F). To facilitate intracellular delivery, we fused a cell-penetrating peptide (CPP) to ACET-1 and ACET-2 to generate CPPtat-1 and CPPtat-2, respectively (Fig. 7G). Functional assays showed that CPPtat-1 markedly enhanced TFCP2 transcriptional activity, whereas CPPtat alone or CPPtat-2 had no effect (Supplementary Fig. S7A-B). Drug sensitivity assays indicated that CPPtat-1 substantially restored erlotinib sensitivity in resistant cells (Fig. 7H). CCK8 assays and colony formation assays further demonstrated that CPPtat-1 improved the response of BxPC-3-R and PANC-1-R cells to erlotinib (Supplementary Fig. S7C-F). This effect was further validated in the PDO-R model (Fig. 7I).

To further assess the in *vivo* efficacy of CPPtat-1, we synthesized its D-enantiomer to improve in *vivo* stability[35, 36]and demonstrated that it markedly potentiates the therapeutic effect of erlotinib. In a KPC mouse model bearing spontaneous, erlotinib-resistant pancreatic tumors, systemic administration of CPPtat-1 combined with erlotinib significantly reduced tumor burden and prolonged overall survival (Fig. 7J and K). IHC analysis confirmed that the combination treatment markedly decreased Ki67 expression in resistant tumors (Fig. 7L and M). Western blot analysis of tumor tissues revealed that CPPtat-1 significantly increased TFCP2 acetylation at K256 and upregulated NDRG1 expression, while concomitantly reducing EGFR levels and indirectly suppressing HDAC1 (Fig. 7N). In PDX-R models, combination therapy with CPPtat-1 further demonstrated its capacity to reverse resistance and corroborated in *vivo* the pivotal role of the HDAC1-TFCP2-NDRG1-EGFR positive feedback axis in driving erlotinib resistance (Fig. 7O-Q and Supplementary Fig. S7G). Collectively, these findings demonstrate that CPPtat-1 competitively blocks TFCP2 deacetylation, restoring its transcriptional activity, and disrupts the HDAC1-TFCP2-NDRG1-EGFR positive feedback signaling axis, thereby effectively reversing erlotinib resistance in PDAC. CPPtat-1 may serve as a potential sensitizer to overcome erlotinib resistance in pancreatic cancer.

## Discussion

EGFR inhibitors like erlotinib are used to treat multiple cancers, but resistance remains a major clinical challenge with incompletely understood mechanisms. Previous studies have demonstrated that adiponectin-2 (LCN2) is upregulated in oral squamous cell carcinoma (OSCC) and is closely linked to EGFR resistance. LCN2 interacts with EGFR, promoting its phosphorylation, activation, and recycling, which subsequently activates the EGFR-MEK-ERK signaling pathway, driving resistance^37^. In NSCLC, secondary mutations in EGFR, such as T790M and C797S, are key mechanisms of resistance to EGFR inhibitors^38^-^41^. Additionally, research has shown that BPTF modulates the c-MYC/PLCG1/pErk signaling axis, affecting gastric cancer (GC) responses to erlotinib^42^. Moreover, PSMD9 contributes to hepatocellular carcinoma (HCC) progression and induces resistance to erlotinib by inhibiting c-Cbl-mediated EGFR ubiquitination^43^. In this study, using a PDAC cell model of acquired erlotinib resistance, combined with transcriptomic sequencing and drug sensitivity analysis, we identified the upregulation of HDAC1 as a key factor driving resistance to erlotinib (Fig. 1). HDAC1 inhibitors may offer promising targeted therapies for PDAC, particularly in cases resistant to EGFR inhibitors. These findings provide new insights and potential therapeutic targets for overcoming resistance to targeted therapies (Fig. 2).

TFCP2, a key transcription factor, plays a dual role in tumorigenesis and development. Although it has primarily been identified as an oncogene, promoting tumorigenesis and metastasis in various cancers, including liver, colorectal, cervical, and breast cancers^44^-^47^, several studies have also reported tumor-suppressive effects of TFCP2 in malignancies such as melanoma, lung cancer, breast cancer, and glioma^48^-^51^. For instance, TFCP2 directly binds to the promoter of the tumor suppressor gene DAPK, enhancing its transcription^49^. Furthermore, TFCP2 undergoes various post-translational modifications, including phosphorylation and methylation, which modulate its DNA-binding efficiency and regulate tumor progression^52^-^54^. This study first identifies HDAC1 as a pivotal epigenetic driver of erlotinib resistance in PDAC. HDAC1 deacetylates TFCP2 at lysine 256 (K256), attenuating its transcriptional activity and reducing NDRG1 expression, which in turn lifts EGFR suppression and activates EGFR-TKI resistance pathways. Furthermore, EGFR-driven tyrosine phosphorylation stabilizes HDAC1 by preventing UPS-mediated degradation, creating a self-perpetuating feedback loop that sustains the resistant state. Collectively, our findings reveal a novel HDAC1-TFCP2-NDRG1 regulatory axis and identify HDAC1 and TFCP2 as promising therapeutic targets for overcoming resistance to EGFR inhibitors.

Developing effective combination therapies is crucial for overcoming drug resistance. Studies suggest that antioxidant agents, statins, and IL-8-targeted therapies may provide improved treatment options for metastatic colorectal cancer (CRC) patients with EGFR resistance^55^. Additionally, molecules such as BPTF, LCN2, and PHGDH have been linked to erlotinib resistance, and targeting these molecules holds promise for reversing resistance in gastric cancer, oral cancer, and lung adenocarcinoma, demonstrating significant therapeutic potential^37^, ^42^, ^56^. Although erlotinib's efficacy in PDAC is limited, various combination strategies have been explored to enhance its anti-tumor activity. These strategies include the combined inhibition of EGFR and C-RAF^57^, the use of erlotinib with MEK1/2 inhibitors^58^, and the combination of the EGFR/IGF-1R dual-target antagonist MK-0646 with gemcitabine^59^. Moreover, while non-selective HDAC inhibitors (HDACi) have been FDA-approved for cancer treatment^60^, their broad-spectrum inhibition is often associated with high toxicity, limiting their clinical applicability^61^, ^62^.

In this study, we screened and validated the HDAC1 inhibitor mocetinostat using multiple cell and animal models, demonstrating its significant reversal of erlotinib resistance in PDAC (Fig. 2). Furthermore, we identified acetylation at lysine K256 of TFCP2 as a critical modification in the resistance mechanism. Based on this finding, we developed a bioactive TFCP2-derived peptide that competitively blocks the deacetylation of TFCP2 by endogenous HDAC1. This strategy preserves TFCP2's transcriptional activity, enhances its regulation of NDRG1, and ultimately reverses the resistant phenotype in PDAC cells (Fig. 7).

The therapeutic potential of competitive-binding peptides has been supported by recent preclinical studies demonstrating robust efficacy and favorable safety profiles across diverse disease models^63^, ^64^. To enhance translational feasibility, we synthesized the TFCP2-K256 peptide using D-amino acids. Compared with conventional L-peptides, D-enantiomeric peptides exhibit markedly improved proteolytic stability, extended plasma half-life, enhanced bioavailability, and reduced immunogenicity, while retaining high target-binding affinity^35^. These pharmacokinetic advantages are critical for maintaining sustained therapeutic activity and minimizing off-target toxicity in *vivo*. Collectively, our approach establishes a strategic framework for targeting protein post-translational modifications, offering a theoretical and practical foundation for next-generation drug development.

Several limitations warrant consideration. First, our conclusions are mainly derived from cell lines and animal models used to validate the HDAC1-TFCP2-NDRG1 axis. While these models provide high controllability, they cannot fully replicate the complex tumor microenvironment—including interactions among immune cells, fibroblasts, and stromal components—or the complete physiological context in humans. Thus, the precise role and clinical relevance of this axis in PDAC patients remain to be established. Moreover, the limited availability of clinical samples from erlotinib-resistant patients constrains our ability to directly validate the therapeutic efficacy and predictive value of our findings. Second, additional regulatory mechanisms and HDAC1-mediated pathways may also contribute to resistance, warranting further investigation. Finally, although we employed Mocetinostat to target HDAC1, current small-molecule inhibitors lack absolute isoform specificity. As a Class I HDAC inhibitor, Mocetinostat exhibits high affinity for HDAC1 but retains partial activity against HDAC2 and HDAC3, representing a persistent technical challenge in the field. Nevertheless, siRNA-mediated knockdown and low-dose Mocetinostat treatments in our study strongly indicate that HDAC1 is the primary mediator of erlotinib resistance, while the possible contribution of other Class I HDACs to the observed phenotype cannot be fully excluded.

In summary, we identified HDAC1 as a pivotal regulator of erlotinib resistance in PDAC. Mechanistically, HDAC1 deacetylates TFCP2 at lysine 256 (K256), suppressing its transcriptional activity and downregulating NDRG1, which in turn lifts EGFR inhibition and activates EGFR-TKI resistance pathways, contributing to acquired erlotinib resistance in PDAC. Moreover, EGFR-mediated tyrosine phosphorylation stabilizes HDAC1 by preventing its UPS-dependent degradation, establishing a self-reinforcing feedback loop that maintains the resistant phenotype. Further studies demonstrated that pharmacological inhibition of HDAC1 or competitively restoring TFCP2 acetylation can significantly reverse the erlotinib-resistant phenotype, providing a theoretical basis and a potential path for clinical translation to overcome EGFR-targeted therapy resistance in PDAC.

## Materials and Methods

### Cell lines and cell culture

HEK293T, BxPC-3, and PANC-1 cell lines used in this study were obtained from Procell Life Science & Technology Co., Ltd. (Wuhan, China). All cell lines were authenticated by short tandem repeat (STR) profiling and routinely tested for mycoplasma contamination using the Lookout Mycoplasma PCR Detection Kit (Sigma-Aldrich). Cells were cultured in Dulbecco's Modified Eagle Medium (DMEM) (Gibco, USA) supplemented with 10% fetal bovine serum (FBS) (Gibco, USA) and maintained at 37 °C in a humidified incubator with 5% CO₂.

### Animals

Six-week-old immunocompromised NOG mice (NOD.Cg-Prkdcscid Il2rgtm1Sug/Jic) were obtained from Vital River Laboratories (Beijing, China) and used to establish patient-derived xenograft (PDX) models. Six-week-old, sex-matched transgenic KPC mice (LSL-KrasG12D/+; LSL-Trp53R172H/+; Pdx-1-Cre) were obtained from Cyagen (Suzhou, China). Four-week-old, sex-matched BALB/c-nu mice were obtained from Shulaibao (Wuhan) Biotechnology Co., Ltd. All animals were maintained under specific pathogen-free (SPF) conditions at Tongji Medical College, Huazhong University of Science and Technology.

### Microbial strains

Competent Escherichia coli strains DH5α or BL21 (DE3) were transformed and plated on LB agar containing 100 μg/mL ampicillin, followed by incubation at 37 °C for plasmid selection.

### Plasmid transfection

Transfection was performed according to the manufacturer's instructions using Lipofectamine 2000 (Thermo Fisher Scientific, Cat#11668030) or Neofect (Renjiu Bio Technology, Cat#TF20121201). Briefly, 2-5 μL of transfection reagent was mixed with 1 μg of plasmid DNA. Both reagents were diluted separately in Opti-MEM medium (Gibco, Cat#11058021), combined, and incubated at room temperature for 5 min to allow complex formation. DNA-lipid complexes were subsequently added to cultured cells.

### RNA interference

Control and gene-specific shRNA lentiviral constructs were obtained from Sigma-Aldrich. The packaging plasmids psPAX2 and pMD2.G were obtained from Addgene and co-transfected with shRNA constructs into HEK293T cells using a lentiviral packaging system. After 24 h of incubation, culture medium was replaced, and viral supernatants were collected by filtration. Viral particles were used to transduce cancer cells in the presence of 6 μg/mL polybrene (Beyotime, Cat#C0351) as a transduction enhancer. Target cells were harvested 48 h after puromycin selection. shRNA and siRNA sequences are listed in Supplementary Table S1.

### CRISPR/Cas9-mediated gene knockout

Oligonucleotides targeting NDRG1 were annealed and cloned into GV708 vectors (Genechem) via restriction enzyme-mediated subcloning. The sgRNA expression vectors were co-transfected with the packaging plasmids pMDG.1 and psPAX2 into HEK293T cells using Lipofectamine 2000, according to the manufacturer's protocol. Culture medium was replaced 24 h after transfection, and viral supernatants were collected 72 h later. Viral particles were concentrated by ultracentrifugation at 80000 × g for 2 h at 4 °C. Transduced cells expressing sgRNA were selected with puromycin (Beyotime, Cat# ST551) at optimized concentrations for 5-7 days to establish stable cell lines. sgRNA sequences used for NDRG1 knockout via CRISPR/Cas9 are listed in Supplementary Table S1.

### Quantitative real-time PCR (qRT-PCR)

Total RNA was extracted using TRIzol reagent (Thermo Fisher Scientific, Cat#15596018). RNA concentration and purity were assessed using a NanoDrop 2000 spectrophotometer (Thermo Fisher Scientific). Reverse transcription was performed using the PrimeScript™ RT Reagent Kit (Takara Bio Inc, Cat#RR037A) according to the manufacturer's instructions. Quantitative real-time PCR (qRT-PCR) was performed using TB Green^TM^ Fast qPCR Mix (Takara Bio Inc, Cat#RR430A) on an iCycler QTX detection system (Bio-Rad). Relative gene expression was calculated using the 2^-ΔΔCT^ method, with GAPDH as the internal control. Primer sequences are listed in Supplementary Table S1.

### Co-immunoprecipitation and western blot analysis

For Co-IP, cells were harvested and lysed on ice for 30 min in IP buffer (Beyotime, Cat#P0013), followed by centrifugation at 12000 rpm for 10 min at 4 °C. The supernatant was incubated overnight at 4 °C with the primary antibody or control IgG in the presence of protein A/G agarose beads (Beyotime, Cat#P2055). The beads were washed at least six times with IP buffer on ice, then resuspended in loading buffer and boiled prior to western blot analysis. Protein concentration of the supernatant was determined using the BCA protein assay (Beyotime, Cat#P0012S). Equal amounts of protein were mixed with loading buffer (Beyotime, Cat#P0015), heated for 10 min, separated by SDS-PAGE, and transferred onto a nitrocellulose membrane (Millipore, Cat#IPVH00005). Membranes were blocked with 5% milk in 1× TBST for 1 h at room temperature, followed by overnight incubation with primary antibodies at 4 °C. After three washes with 1×TBST, membranes were incubated with HRP-conjugated secondary antibodies for 1 h at room temperature. Protein bands were detected using ECL detection reagents (Meilunbio, Cat#MA0186) and visualized with a ChemiDoc XRS imaging system (Bio-Rad).

### ChIP-qPCR

ChIP assays were performed on pancreatic cancer cells according to the manufacturer's instructions, using the Pierce Magnetic ChIP Kit (Thermo Fisher Scientific, Cat#26157). Briefly, cells were crosslinked with 1% formaldehyde for 10 min at room temperature, and the reaction was quenched with 125 mM glycine for 5 min. Cells were then harvested and lysed, and chromatin was digested enzymatically according to the kit protocol. Chromatin immunoprecipitation (ChIP) was performed using protein G magnetic beads and a specific antibody, with ~5 μg of antibody per 25 μg of chromatin. Normal rabbit IgG was used as a negative control. The PCR Kit (Promega) was used to amplify purified input and immunoprecipitated DNA. Purified DNA was analyzed by quantitative PCR (qPCR) to assess promoter and enhancer regions. Primer sequences and antibodies used are listed in Supplementary Table S1.

### Dual-luciferase reporter assay

BxPC-3 and PANC-1 cells were co-transfected with luciferase reporter constructs containing gene promoters cloned into the pGL3-basic vector (Promega) and a Renilla luciferase control plasmid (phRL-TK, Genechem). Transcriptional activity was measured using the Dual-Luciferase Reporter Assay System (Promega), with firefly luciferase activity normalized to Renilla luciferase activity to control for transfection efficiency.

### Peptide synthesis

All peptides were synthesized by Guoping Pharmaceutical Co., Ltd. (Hefei, China) and purified to >98% purity using high-performance liquid chromatography (HPLC) for both in *vitro* and in *vivo* applications. Peptides used in *vivo* were composed entirely of D-isomer amino acids. For in *vitro* studies, peptides were dissolved in PBS to prepare 10 mM stock solutions. For in *vivo* administration, CPPtat and CPPtat-1 were dissolved in PBS and kept on ice until injection, with all solutions allowed to equilibrate to room temperature immediately prior to use.

### RNA sequencing

RNA sequencing was performed by Haplox Biotechnology (Jiangxi, China). Approximately 30 mg of tissue was ground in liquid nitrogen, and total RNA was extracted using TRIzol^TM^ Reagent (Thermo, #15596018). RNA purity was assessed using a NanoDrop^TM^ One (Thermo, #ND-ONE-W), and RNA concentration was measured with the Qubit™ RNA BR Assay Kit (Invitrogen, #Q10210) on a Qubit^TM^ 3 Fluorometer (Thermo, #Qubit 3.0). RNA integrity was evaluated using RNA ScreenTape and Sample Buffer (Agilent, #5067-5576 and #5067-5577) on an Agilent 4200 TapeStation System. For mRNA library preparation, 0.1-1 μg of total RNA was subjected to poly(A) RNA isolation using the NEBNext® Poly(A) mRNA Magnetic Isolation Module (NEB, #E7490L), followed by library construction with the NEBNext® Ultra^TM^ II mRNA Library Prep Kit for Illumina® (NEB, #E7770L). Library concentration and fragment size distribution were measured using the Qubit^TM^ dsDNA HS Assay Kit (Invitrogen, #Q32851) and D1000 ScreenTape with D1000 reagents (Agilent, #5067-5582 and #5067-5583), respectively. Library molarity was quantified using the KAPA Library Quantification Kit (Illumina, #KK4824) on a QuantStudio^TM^ 3 Real-Time PCR System (Thermo, #A28572). Sequencing was performed on a NovaSeq 6000 platform using the NovaSeq S4 Reagent Kit (Illumina) according to the manufacturer's instructions.

Raw RNA-seq reads (FASTQ format) were processed using fastp for quality control, adapter trimming, and per-read filtering to generate high-quality clean reads. Clean reads were aligned to the reference genome using HISAT2 v2.1.0 with default parameters. Gene-level read counts were quantified using featureCounts, and expression levels were normalized as FPKM (fragments per kilobase of transcript per million mapped reads). Differential gene expression between groups with biological replicates was analyzed using the DESeq2 R package (v1.18.1), which models read counts with a negative binomial distribution. P-values were adjusted for multiple testing using the Benjamini-Hochberg method, and genes with |log_2_(fold change)| > 1 and adjusted P < 0.05 were considered differentially expressed.

### GST pulldown assay

The GST-tagged TFCP2 protein was expressed in E. coli BL21(DE3) cells harboring the GST-TFCP2 plasmid. Expression was induced with 0.5 mM IPTG at 16 °C for 16 h. The bacterial cells were harvested and lysed, and the GST-TFCP2 fusion protein was affinity-purified using glutathione-Sepharose beads. For the pull-down assay, cells were lysed on ice for 30 min in IP lysis buffer (Beyotime Biotechnology, Cat# P0013), followed by brief sonication. Purified GST-TFCP2 bound to beads was incubated with the cell lysates overnight at 4 °C. The beads were subsequently washed eight times on ice with ice-cold binding buffer (20 mM Tris, 100 mM NaCl, 1 mM EDTA, 5% glycerol, 1 mM DTT, 1 mM PMSF, and 1 mM benzamidine). Bound proteins were then eluted using ice-cold IP lysis buffer and subjected to western blot analysis.

### Mass spectrometry

To identify novel HDAC1-interacting proteins, PANC-1 cells were subjected to immunoprecipitation using an anti-HDAC1 antibody and protein A+G agarose (Beyotime, Cat#P2055) at 4 °C. The immunoprecipitated complexes were subsequently analyzed by LC-MS/MS using a Thermo Ultimate 3000 liquid chromatography system coupled to a Q Exactive Plus high-resolution mass spectrometer at SpecAlly Life Technology Co., Ltd. (Wuhan, China). Raw data were processed using MaxQuant (v1.6.6) with the Andromeda search engine. Database searching was performed against the UniProt human proteome reference database. Proteins and peptides identified with a false discovery rate (FDR) of 1% were retained for further analysis.

### Cell surface biotinylation assay for receptor internalization

To monitor receptor internalization, a cell surface biotinylation approach was employed. Cells were first serum-starved overnight to synchronize receptor activity. Surface proteins were labeled by incubating cells on ice with 0.5 mg/ml sulfo-NHS-SS-biotin in D-PBS (pH 7.4) for 2 hours. Excess and unreacted biotin were quenched by washing cells multiple times with 50 mM NH_4_Cl on ice. Receptor internalization was then induced by stimulating cells with EGF at 37°C for 0, 5, 15, and 30 minutes. Following stimulation, cells were rapidly cooled to 4°C and treated with 100 mM MESNA for 10 minutes to selectively remove biotin from proteins remaining on the cell surface, while internalized biotinylated proteins were protected from reduction. Parallel samples without MESNA treatment were included to assess total surface biotinylation. Free thiols were subsequently blocked by incubating cells on ice with iodoacetamide. Cells were lysed in appropriate lysis buffer, and lysates were clarified by centrifugation. A small portion of the lysate was reserved for total protein measurement, and the remaining lysate was incubated with streptavidin-conjugated magnetic beads at 4°C overnight to capture biotinylated proteins. Beads were collected, and bound proteins were eluted in SDS-PAGE sample buffer by heating. Receptor levels were analyzed by Western blotting, and internalization efficiency was quantified as the ratio of internalized biotinylated receptor to total biotinylated receptor, expressed as a percentage.

### Isolation of lysosomal proteins

Lysosomal fractions were obtained using a commercial lysosome enrichment kit (Thermo Fisher Scientific) following the manufacturer's protocol with minor procedural adjustments. In brief, approximately 5 × 10^7^ cells were harvested and lysed using the buffers provided in the kit. Cell disruption was further facilitated by sonication to ensure efficient release of intracellular organelles. The resulting lysates were then subjected to ultracentrifugation at 145000×g at 4°C to separate and enrich the lysosomal protein fraction. The collected samples were subsequently mixed with Laemmli sample buffer, denatured, and analyzed by Western blotting to detect proteins of interest.

### Immunohistochemistry

PDAC tissue samples used in this study were obtained from Wuhan Union Hospital, with informed consent obtained from all patients. Pancreatic cancer tissue microarrays were provided by Bioaitech. Immunohistochemical staining intensity and the proportion of positive cells were quantified using ImageJ software. Staining intensity was graded as 0 (negative), 1 (weak), 2 (moderate), or 3 (strong). The H-score was calculated using the formula: H-score = (% of cells at each staining intensity × corresponding intensity score), and the values were summed to generate the final score. All evaluations were independently performed by two pathologists blinded to the experimental conditions. Details of the antibodies used are provided in Supplementary Table S2. Immunohistochemical staining was performed by BIOSSCI Biotech Co., Ltd. (Hubei, China).

### Colony formation assay

Cells (500 cells per well) were seeded into 6-well plates containing 2 mL of DMEM supplemented with 10% FBS. After incubation for 2 weeks, colonies were fixed with 4% paraformaldehyde for 30 min, stained with 1% crystal violet staining solution (Beyotime, #C0121) for 20 min, and subsequently washed three times with PBS.

### CCK-8 assay

Cells were seeded into 96-well plates at a density of 3000 cells per well in 200 μL of DMEM supplemented with 10% FBS and cultured for 5 days. One hour before the end of the incubation period, 20 μL of CCK-8 reagent (Beyotime, #C0037) was added to each well according to the manufacturer's instructions. Absorbance at 450 nm was measured using a microplate reader to evaluate cell viability.

### 3D cell culture

Cells were cultured in a three-dimensional (3D) system using Matrigel (Cat#356234, Corning). Briefly, cells were trypsinized, centrifuged, and resuspended in DMEM at a final density of 3×10^5^ cells mL^-1^. The cell suspension was then seeded into 24-well plates pre-coated with Matrigel and incubated at 37 °C for 30 mins to allow gel solidification. Subsequently, DMEM supplemented with 10% Matrigel was added, and cells were cultured for 7 days. The culture medium was refreshed every 2 days with Matrigel-containing DMEM. Cell growth was assessed using the CCK-8 assay.

### Establishment of erlotinib-resistant cell lines

Erlotinib-resistant BxPC-3 and PANC-1 cell lines were generated using a stepwise dose-escalation strategy. Baseline sensitivity of parental cells to erlotinib was determined by CCK-8 assay, yielding IC_50_ values of 14.11 μM for BxPC-3 and 5.27 μM for PANC-1. To induce acquired resistance, cells were initially exposed to 2 μM erlotinib for BxPC-3 and 1 μM for PANC-1. Cells were maintained in complete medium containing erlotinib, with the medium refreshed every 2-3 days. Stable proliferation at each concentration was defined as cell growth reaching approximately 80% confluence with a doubling time comparable to untreated parental controls. Once cells met these criteria, the erlotinib concentration was increased stepwise by 20-50% of the previous concentration. Each concentration stage was maintained for 1-2 weeks, or until growth kinetics resembled those of untreated cells. This stepwise escalation protocol was continued for approximately four months. Dose-response assays confirmed the establishment of resistant sublines, with IC_50_ values increasing to 75.52 μM for BxPC-3 and 33.65 μM for PANC-1. The resistance index (RI), calculated as the ratio of IC_50_ of resistant cells to parental cells, was 5.35 for BxPC-3 and 6.38 for PANC-1.

### Spontaneous pancreatic cancer model

KPC transgenic mice (SL-KrasG12D/+; LSL-Trp53R172H/+; Pdx-1-Cre; 6 weeks old, sex-matched) were purchased from Cyagen (Suzhou, China) and maintained in SPF conditions. At 6 weeks of age, mice received daily oral administration of erlotinib (100 mg/kg, Selleck Chemicals, Cat# S7786). Tumor volume was monitored by high-frequency small-animal ultrasound imaging every three days. The in *vivo* resistant model was deemed successfully established when a treatment-induced initial regression of the tumor was followed by subsequent relapse. To investigate the reversal of erlotinib resistance by mocetinostat, at 16 weeks, mice were randomly assigned to four groups (n=5 per group) and treated with vehicle, mocetinostat (80 mg/kg, Selleck Chemicals, Cat# S1122, p.o., q.d.), erlotinib (100 mg/kg, p.o., q.d.), or their combination. Tumor growth was monitored twice per week. After 4 weeks of treatment, mice were euthanized, and their pancreases were harvested for subsequent analysis. To investigate the reversal of erlotinib resistance by CPPtat-1, at 16 weeks, mice were randomly assigned to four groups (n = 5 per group) and treated with CPPtat (Vehicle), CPPtat-1 (100 mg/kg, i.v., q.d.), erlotinib (100 mg/kg, p.o., q.d.), or their combination. Tumor growth was monitored twice per week. After 4 weeks of treatment, mice were euthanized, and their pancreases were harvested for subsequent analysis.

### Patient-derived xenografts (PDX)

The erlotinib-resistant PDX model was generated by subcutaneous implantation of pathologically confirmed PDAC surgical specimens, minced into small fragments, into NOG mice (F0 generation). Once tumors reached ~100 mm³, daily oral erlotinib treatment commenced. Successful resistance was defined by initial tumor regression followed by regrowth. Tumors were passaged at ~800 mm³ and serially propagated to F3 to generate a stable erlotinib-resistant PDX model. At 100 mm³, F3 tumors were randomized into four groups: vehicle, erlotinib (100 mg/kg, p.o., daily), mocetinostat (80 mg/kg, p.o., daily), or the combination. To assess reversal of erlotinib resistance by CPPtat-1, mice received CPPtat (vehicle), CPPtat-1 (100 mg/kg, i.v., daily), erlotinib (100 mg/kg, p.o., daily), or their combination. Treatments continued for 30 days, after which mice were euthanized and PDX tissues harvested for IHC analysis. Tumor volumes were measured every three days and calculated as: volume (mm^3^) = (width)^2^ × length × 1/2.

### Mice xenograft model

Four-week-old BALB/c-nu mice were randomly assigned to four groups (n = 5 per group). Mice were subcutaneously inoculated with 5 × 10⁶ PANC-1-R (NDRG1-KO) cells. When tumor volumes reached approximately 50 mm³, mice were treated with vehicle, mocetinostat (80 mg kg^-1^, p.o., once daily), erlotinib (100 mg kg^-1^, p.o., once daily), or the combination of both agents. Tumor volumes were measured every three days. After 30 days of treatment, tumors were excised, weighed, photographed, and subjected to further analyses.

### Generation of erlotinib-resistant PDAC organoids

Primary PDAC organoids were exposed to erlotinib through stepwise dose escalation to establish drug-resistant lines. Organoids were initially treated with a sublethal concentration of erlotinib. Following adaptation and recovery of stable growth, the drug concentration was gradually increased to a final concentration corresponding to 5×IC_50_. The culture medium was refreshed weekly, and organoid growth was continuously monitored throughout the selection process.

### Statistical analysis

Statistical analyses were performed using GraphPad Prism 9.5. Comparisons between two groups were conducted using an unpaired two-tailed Student's t-test. For comparisons involving three or more groups, one-way or two-way ANOVA was applied as appropriate, followed by Tukey's post hoc test for multiple comparisons. A P value < 0.05 was considered statistically significant. Data are presented as mean ± standard deviation (SD). ns, not significant; *P < 0.05; **P < 0.01; ***P < 0.001.

## Supplementary Material

Supplementary figures and tables.

## Data Availability

The data supporting the findings of this study are available from the corresponding author (Dianyun Ren, rendianyun@hust.edu.cn) upon reasonable request.
