# Supplementary material for: HDAC1 Regulates Acquired Resistance to EGFR Inhibitors through the TFCP2-NDRG1 Signaling Axis in Pancreatic Cancer
Source: Int J Biol Sci. 2026 Apr 8;22(8):4346–66. doi: 10.7150/ijbs.131003 (PMC13137961; doi:10.7150/ijbs.131003)

Supplementary materials for

HDAC1 Regulates Acquired Resistance to EGFR Inhibitors through the TFCP2–NDRG1 Signaling Axis in Pancreatic Cancer

Taoyu Chen, Yuxuan Li, Yan Sun, Qixun Fu, Heshui Wu, Dan Li, Dianyun Ren

The file includes:

- Supplementary figures and figure legends
- Supplementary Table S1. Sequence of primers and gene specific shRNAs, siRNAs and gRNAs
- Supplementary Table S2. Key Resources
- Supplementary Table S3. Clinical information of patients in TMA

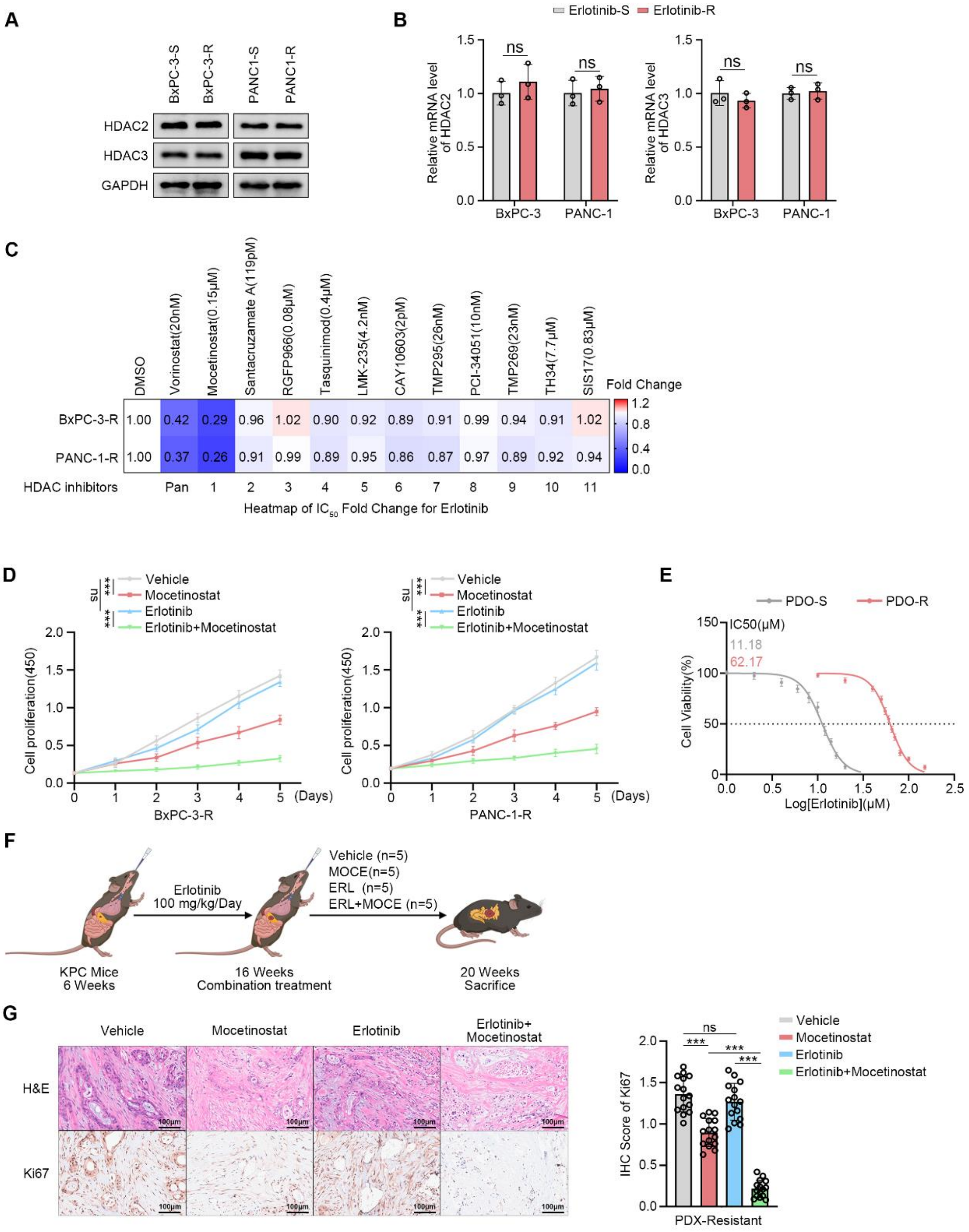

Supplementary Fig. S1 Co-silencing of HDAC1 reverses erlotinib resistance. (A and B) Protein and mRNA expression levels of HDAC2 and HDAC3 in BxPC-3 and PANC-1 cells were examined by Western blot and qRT-PCR, respectively. Data are presented as mean ± SD (n = 3). Data are mean ± SD. n=3, ns, not significant. **(C)**

Heatmap depicting fold changes in erlotinib IC_{50} following treatment with the indicated drugs. **(D)** Cell viability of BxPC-3-R and PANC-1-R cells treated with DMSO, mocetinostat (0.15 μM), erlotinib (20 μM), or their combination, measured by CCK-8 assay. **(E)** IC_{50} values of erlotinib in sensitive and resistant pancreatic tumor organoids. **(F)** Schematic illustrating the generation of an erlotinib-acquired resistant KPC mouse model and the corresponding treatment regimen. Mice were administered erlotinib (100 mg/kg, p.o., q.d.), mocetinostat (80 mg/kg, p.o., q.d.), or their combination as indicated. **(G)** Representative IHC images of tumors (Left) and corresponding quantification of IHC scores (Right). Scale bars=100 μm. n = 5 biologically independent samples; 3 independent IHC quantifications. Data are mean ± SD; ns, not significant; ***$P < 0.001$.

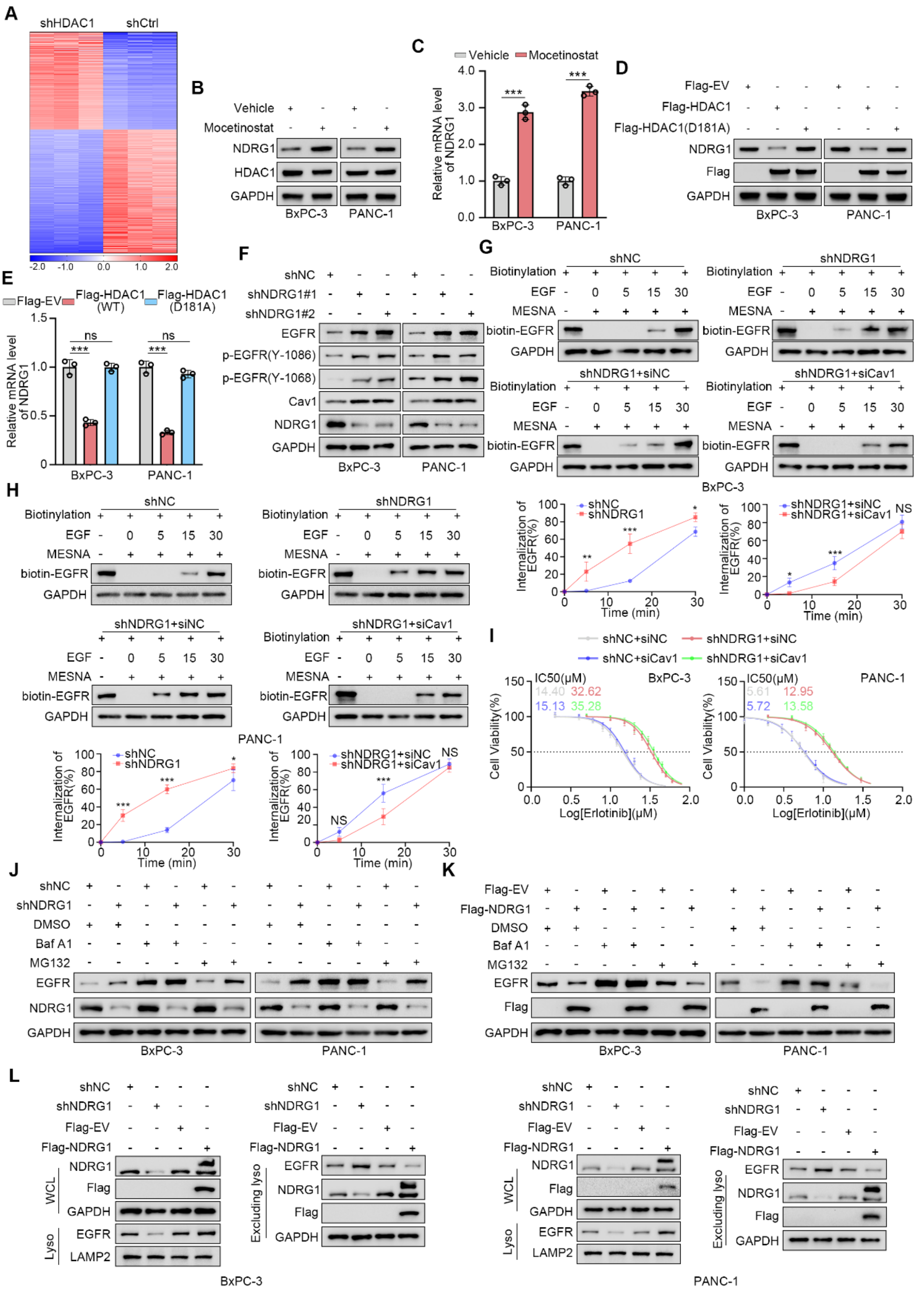

Supplementary Fig. S2 NDRG1 mediates EGFR degradation via the lysosomal pathway. (A) Heatmap showing DEGs from RNA-seq in PANC-1 cells infected with shHDAC1 or shCtrl. **(B and C)** NDRG1 protein (B) and mRNA (C) levels in BxPC-3 and PANC-1 cells treated with vehicle or mocetinostat (0.15 μM, 48 h). n = 3; Data are

mean ± SD; ****P* < 0.001. **(D** and **E)** NDRG1 protein (D) and mRNA (E) levels in BxPC-3 and PANC-1 cells transfected with indicated plasmids. n = 3; Data are mean ± SD; ns, not significant; ****P* < 0.001. **(F)** Western blot analysis of EGFR, p-EGFR, and Cav1 protein levels in BxPC-3 and PANC-1 cells following NDRG1 knockdown. **(G** and **H)** EGFR internalization in PANC-1 and BxPC-3 cells under indicated conditions was assessed using cell surface biotinylation, as described in "Materials and Methods." n = 3; Data are mean ± SD; ns, not significant; **P* < 0.05; ***P* < 0.01; ****P* < 0.001. **(I)** IC_{50} values of PANC-1 and BxPC-3 cells under the indicated conditions., determined by CCK-8 assay. **(J** and **K)** Western blot analysis of EGFR protein levels in PANC-1 and BxPC-3 cells transfected with the indicated shRNAs or plasmids and treated with DMSO, BafA1 (50 nM, 6 h), or MG132 (10 μM, 8 h). **(L)** Cells were transfected with the indicated plasmids, and lysosomes were isolated. Proteins from both the lysosomal and non-lysosomal fractions were extracted for Western blot analysis to assess EGFR levels inside and outside the lysosomes.

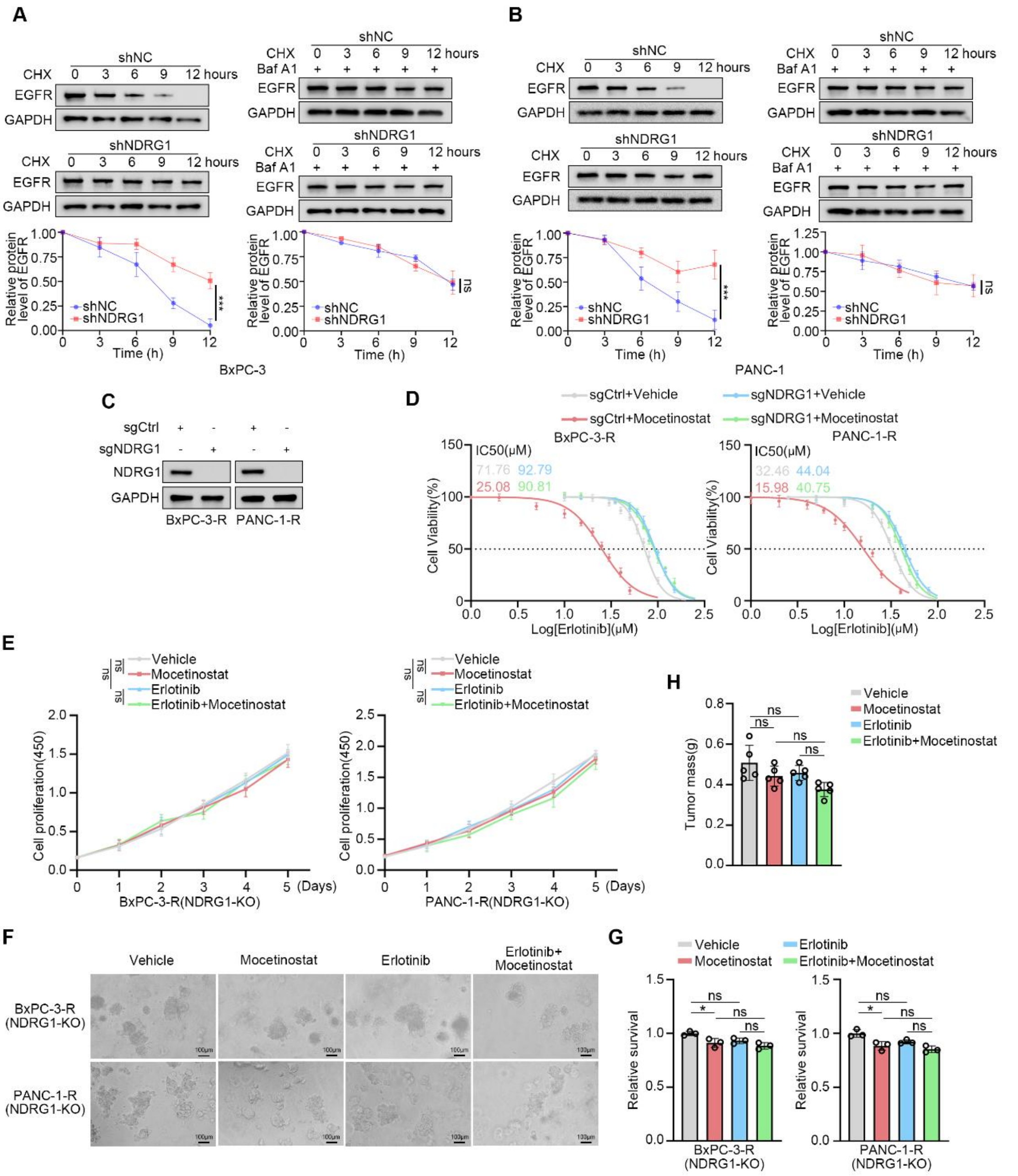

Supplementary Fig. S3 HDAC1 promotes erlotinib resistance by downregulating NDRG1. (A and B) BxPC-3 and PANC-1 cells were transfected with the indicated plasmids and treated with CHX (10 μM) alone or in combination with Baf A1 (50 nM). Cells were harvested at different time points to assess EGFR levels. n = 3; Data are mean ± SD; ns, not significant; **P* < 0.05; ***P* < 0.01; ****P* < 0.001. **(C)** Western blot confirming NDRG1 knockout efficiency in BxPC-3-R and PANC-1-R cells via CRISPR–Cas9. **(D)** Erlotinib IC_{50} in BxPC-3-R and PANC-1-R cells with or without NDRG1 knockout, treated with vehicle, mocetinostat (0.15 μM), erlotinib (20 μM), or

their combination, measured by CCK-8 assay. **(E)** Cell viability of NDRG1-KO BxPC-3-R and PANC-1-R cells under the indicated treatments, measured by CCK-8 assay. **(F and G)** 3D culture assays of NDRG1-KO BxPC-3-R and PANC-1-R cells treated as indicated; quantification shown. n = 3; Data are mean ± SD; ns, not significant; $*P < 0.05$. **(H)** Tumor weights on day 30 in a subcutaneous xenograft model treated with vehicle, mocetinostat (80 mg/kg, p.o., q.d.), erlotinib (100 mg/kg, p.o., q.d.), or combination. n = 5; Data are mean ± SD; ns, not significant.

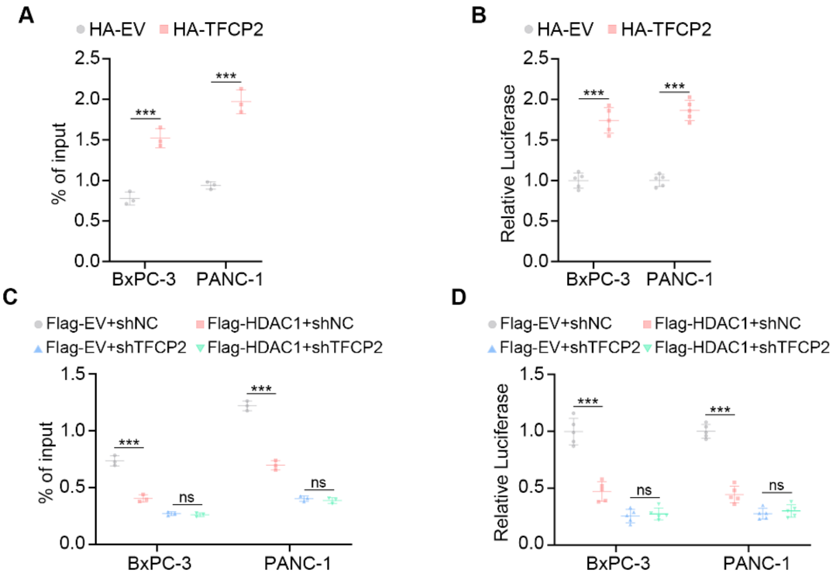

Supplementary Fig. S4 HDAC1 Regulates NDRG1 Expression by Binding to TFCP2. (A) ChIP–qPCR analysis of TFCP2 binding at NDRG1 promoter regions in BxPC-3 and PANC-1 cells transfected with indicated plasmids. n = 3; Data are mean ± SD; ****P* < 0.001. **(B)** Luciferase reporter assays measuring NDRG1 transcriptional activity in BxPC-3 and PANC-1 cells with indicated plasmids. n = 5; Data are mean ± SD; ****P* < 0.001. **(C and D)** BxPC-3 and PANC-1 cells transfected with indicated plasmids were analyzed by ChIP–qPCR (C) for TFCP2 enrichment at NDRG1 promoters and by luciferase reporter assays (D) for NDRG1 transcriptional activity. Luciferase assays n = 5; others n = 3; Data are mean ± SD; ns, not significant; ****P* < 0.001.

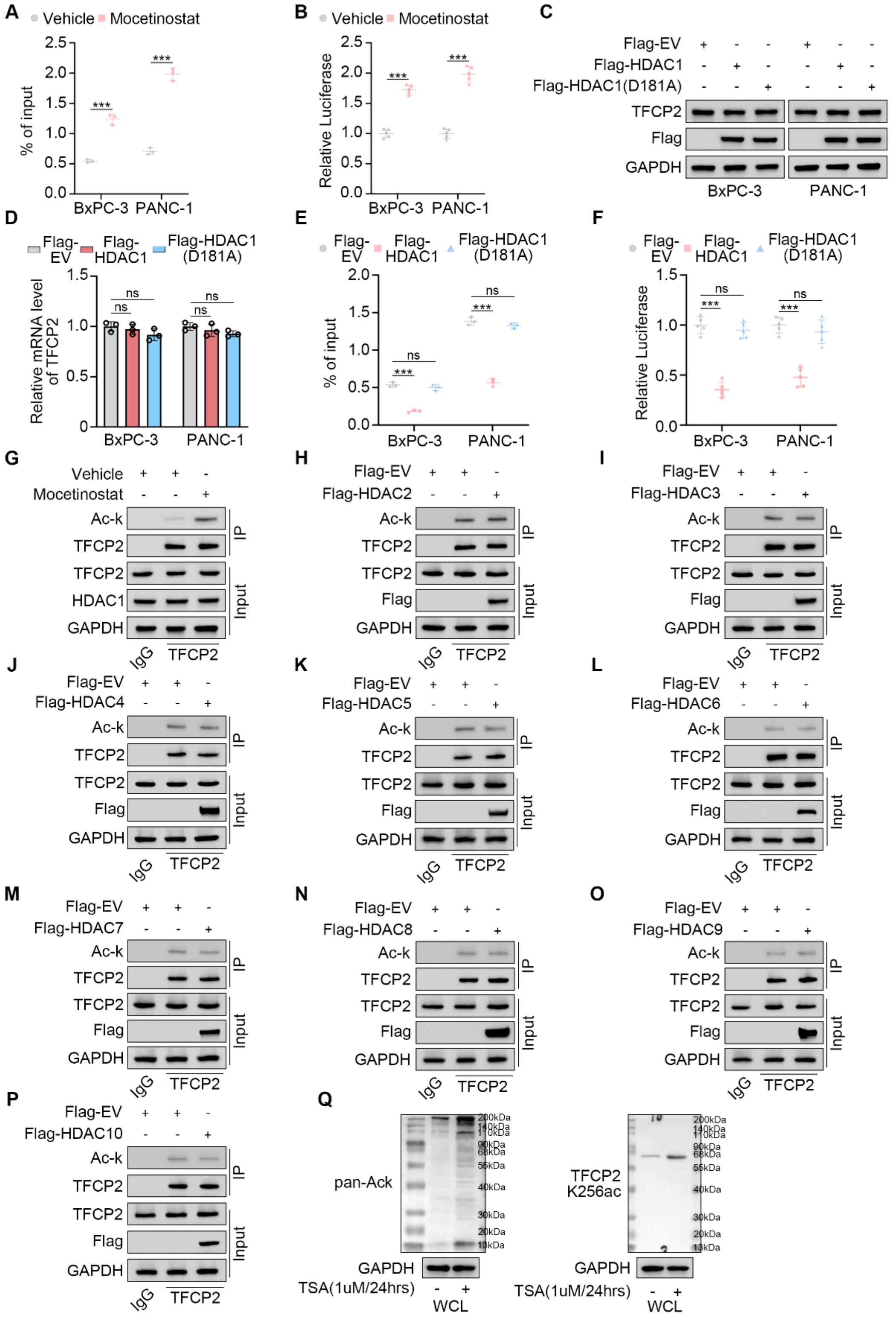

Supplementary Fig. S5 HDAC1 deacetylates TFCP2 at K256 to inhibit transcriptional activity. (A) ChIP–qPCR analysis of TFCP2 enrichment at the NDRG1 promoter in BxPC-3 and PANC-1 cells treated with vehicle or mocetinostat (0.15 μM). n=3, Data are mean ± SD; ns, not significant; $***P < 0.001$. **(B)** Luciferase

reporter assays assessing TFCP2 transcriptional activity in BxPC-3 and PANC-1 cells treated with vehicle or mocetinostat. n=5, Data are mean ± SD; ns, not significant; ***P < 0.001. **(C** and **D)** Western blot (C) and qRT–PCR (D) analyses of TFCP2 protein and mRNA levels in BxPC-3 and PANC-1 cells expressing the indicated plasmids. n = 3; Data are mean ± SD; ns, not significant. **(E)** ChIP–qPCR analysis of TFCP2 binding at the NDRG1 promoter in BxPC-3 and PANC-1 cells expressing the indicated plasmids. n = 3; Data are mean ± SD; ***P < 0.001. **(F)** Luciferase reporter assays evaluating TFCP2 transcriptional activity in cells expressing the indicated plasmids. n = 5; Data are mean ± SD; ***P < 0.001. **(G)** Co-IP detecting acetylated TFCP2 in PANC-1 cells treated with vehicle or mocetinostat (0.15 μM, 48 h). **(H-P)** Co-IP analysis of TFCP2 acetylation in PANC-1 cells transfected with the indicated plasmids. **(Q)** Western blot analysis of whole-cell lysates from PANC-1 cells treated with DMSO or TSA (1 μM, 18 h) using a pan–acetyl-lysine antibody (left) and a TFCP2 K256ac–specific antibody (right).

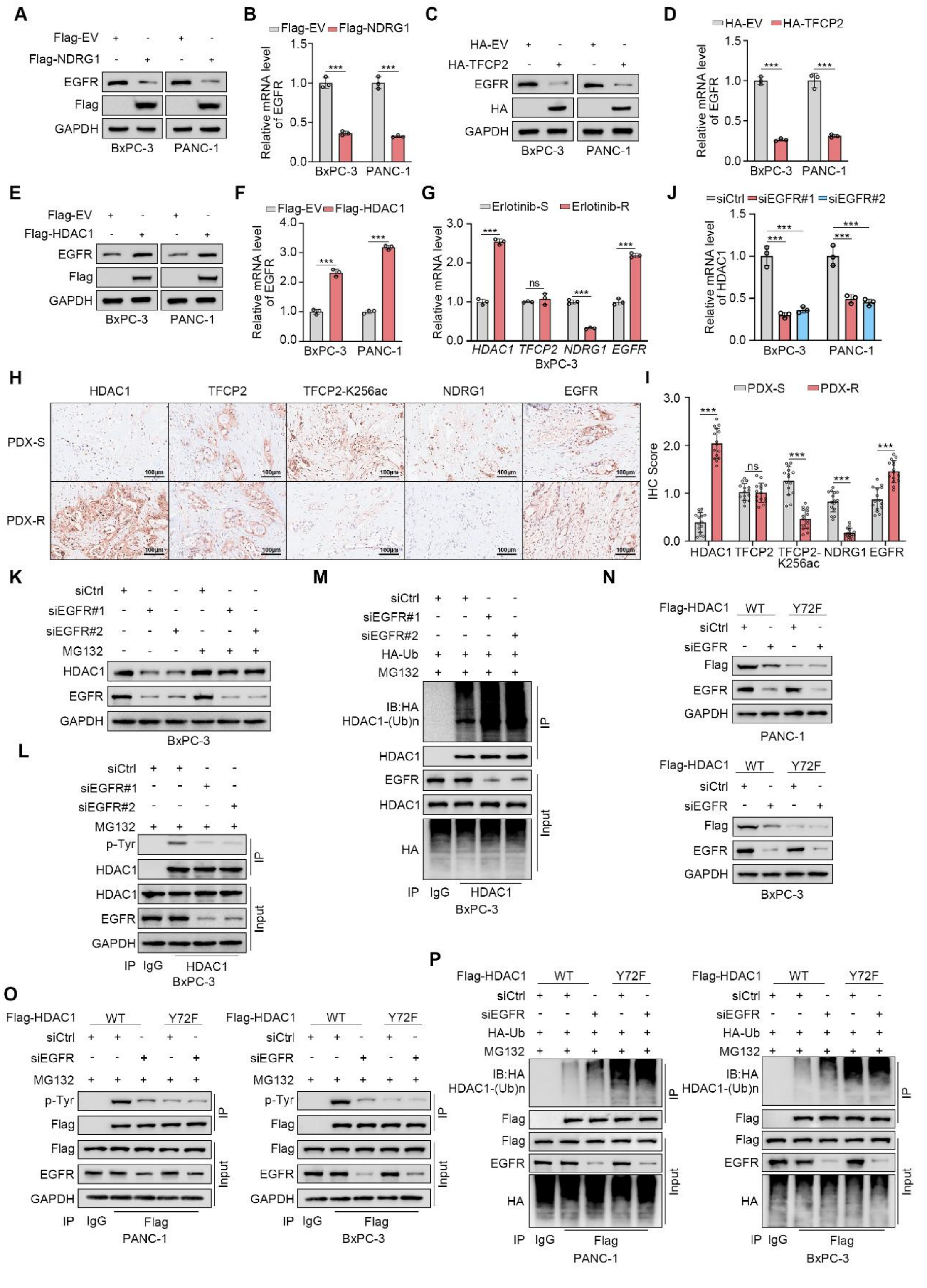

Supplementary Fig. S6 TFCP2 acetylation sustains the HDAC1-TFCP2-NDRG1-EGFR positive feedback loop. (A-F) Western blot and RT-qPCR analyses of EGFR protein and mRNA levels in BxPC-3 and PANC-1 cells transfected with the indicated plasmids. n=3, Data are mean ± SD; ****P* < 0.001. **(G)** RT-qPCR analysis of the

indicated genes in BxPC-3-S amd BxPC-3-R cells. n=3, Data are mean ± SD; ***$P < 0.001$. **(H** and **I)** Representative IHC images of PDX-S and PDX-R tumors (H) and quantification of IHC scores (I). n = 5 biologically independent samples; 3 independent IHC quantifications. Data are mean ± SD; ns, not significant; ***$P < 0.001$. **(J)** RT-qPCR analysis of HDAC1 mRNA levels in BxPC-3 and PANC-1 cells transfected with the indicated siRNAs. **(K)** Western blot of HDAC1 in BxPC-3 cells transfected with the indicated siRNAs and treated with DMSO or MG132 (10 μM, 8 h). **(L)** Co-IP analysis of HDAC1 tyrosine phosphorylation in EGFR-deficient BxPC-3 cells. **(M)** Co-IP of HDAC1 ubiquitination in EGFR-deficient BxPC-3 cells. **(N)** Western blot analysis of Flag-HDAC1 (WT) and Flag-HDAC1 (Y72F) protein levels in PANC-1 and BxPC-3 cells transfected with the indicated siRNAs. **(O** and **P)** Co-IP analysis of Flag-HDAC1 (WT) and Flag-HDAC1 (Y72F) in PANC-1 and BxPC-3 cells under the indicated conditions, showing tyrosine phosphorylation **(O)** and ubiquitination **(P)** levels.

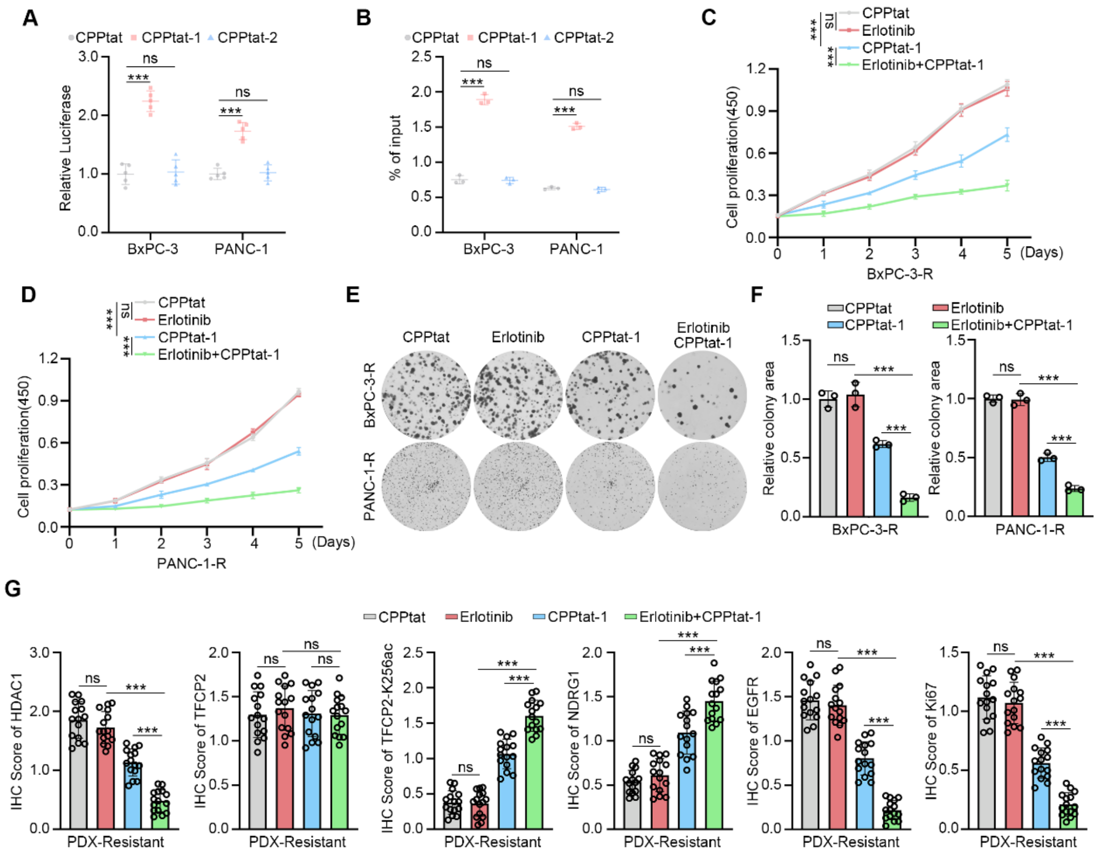

Supplementary Fig. S7 Acetylation of TFCP2 at K256 reverses erlotinib resistance. **(A)** Luciferase reporter assays assessing TFCP2 transcriptional activity in BxPC-3 and PANC-1 cells treated with CPPtat (5 μM), CPPtat-1 (5 μM), or CPPtat-2 (5 μM). n=5, Data are mean ± SD; ns, not significant; ****P* < 0.001. **(B)** ChIP–qPCR analysis of TFCP2 enrichment at the NDRG1 promoter in BxPC-3 and PANC-1 cells treated with CPPtat, CPPtat-1 (5 μM), or CPPtat-2 (5 μM). n=3, Data are mean ± SD; ns, not significant; ****P* < 0.001. **(C and D)** Cell viability of BxPC-3-R and PANC-1-R cells treated with CPPtat, CPPtat-1, erlotinib, or their combination, measured by CCK-8 assay. n=3, Data are mean ± SD; ns, not significant; ****P* < 0.001. **(E and F)** Representative images (E) and quantification (F) of colony formation assays in BxPC-3-R and PANC-1-R cells treated with CPPtat (5 μM), CPPtat-1 (5 μM), erlotinib (20 μM), or their combination. n=3, Data are mean ± SD; ns, not significant; ****P* < 0.001. **(G)** Quantification of IHC scores in PDX-R tumors. n = 5 biologically independent samples; 3 independent IHC quantifications. Data are mean ± SD; ns, not significant; ****P* < 0.001.

Supplementary Table S1.
Sequence of primers and gene specific shRNAs, siRNAs and gRNAs

Gene	Forward		Reverse
GAPDH	RT-qPCR	ACCCAGAAGACTGTGGATGG	TTCAGCTCAGGGATGACCTT
HDAC1	RT-qPCR	CTACTACGACGGGGATGTTGG	GAGTCATGCGGATTCGGTGAG
HDAC2	RT-qPCR	CTGCTACTACTACGACGGTGA	GTCATTTCTTCGGCAGTGGC
HDAC3	RT-qPCR	GAGTTCTGCTCGCGTTACACAG	CGTTGACATAGCAGAAGCCAGAG
TFCP2	RT-qPCR	TCTGGCCGACGAAGTGATTG	ATCAGGAGGCAAACTCGACTC
NDRG1	RT-qPCR	CTCCTGCAAGAGTTTGATGTCC	TCATGCCGATGTCATGGTAGG
EGFR	RT-qPCR	AGGCACGAGTAACAAGCTCAC	ATGAGGACATAACCAGCCACC
NDRG1	Chip-qPCR	CCTCTCCCTTTGCCAGTGAG	CACCTCTAGGACCCAGGACA
**SiRNAs**	**Sequence**		
SiControl	UUCUCCGAACGUGUCACGUTT		
SiHDAC1#1	CAGCGAUGACUACAUUAAAUU		
SiHDAC1#2	GCUUCAAUCUAACUAUCAAAG		
SiHDAC2#1	GGUCAAUAAGACCAGAUAACA		
SiHDAC2#2	AGAAGAUGCUGUUCAUGAAGA		
SiHDAC3#1	AGAAGAUGAUCGUCUUCAAGC		
SiHDAC3#2	GGUAGUGGACUUCUACCAACC		
SiHDAC4#1	GCACAGAAGUGAAGAUGAAGU		

SiHDAC4#2	GGACAGUAAGAAACUUCUAGG
SiHDAC5#1	GGACUGGGACAUUCACCAUTT
SiHDAC5#2	GGCAGAAGCUAGACAGCAAGA
SiHDAC6#1	GUUCUAAGUUGGUCACCAAGA
SiHDAC6#2	GGGUGAUGCUGACUACCUAGC
SiHDAC7#1	GCAGCGUGGUCAAGCAGAAGC
SiHDAC7#2	GCAAGAUCCUCAUUGUAGACU
SiHDAC8#1	GGACGGUACUACAGUGUAAAU
SiHDAC8#2	CCAUGGAGAUGGUGUAGAAGA
SiHDAC9#1	GCUGGUCAUUCAACAGCAACA
SiHDAC9#2	GGUAAUAGGCAAAGAUUUAGC
SiHDAC10#1	GGUUCUGUGUGUUCAACAACG
SiHDAC10#2	ACGGGUUCUGUGUGUUCAACA
SiHDAC11#1	GAGACUUCAUGGACGACAAGC
SiHDAC11#2	AGGUGGAGAGGAACAUCAAGA
SiEGFR#1	GGUGUGUGCAGAUCGCAAAGG
SiEGFR#1	GCAUGUCAAGAUCACAGAUUU
siCAV1	GCAUUUGGAAGGCCAGCUUTT
**shRNAs**	**Sequence**
shHDAC1#1	CGGTTAGGTTGCTTCAATCTA
shHDAC1#2	GCTGCTCAACTATGGTCTCTA
shTFCP2#1	GCTCTTGTGGTACACACAGAT
shTFCP2#2	CCTTCCTATGAGACAACCATA
shNDRG1#1	GCACATTGTGAATGACATGAA
shNDRG1#2	GCCTACATCCTAACTCGATTT
**gRNAs**	**Sequence**
gNDRG1	TATCAACGTGAACCCTTGTG

Supplementary Table S2. Key Resources

Reagent or Resource	Source	Identifier
**Antibodies**		
Rabbit polyclonal anti-GAPDH	Proteintech	Cat# 10494-1-AP<br>RRID: AB_2263076
Rabbit polyclonal anti-HDAC1	Proteintech	Cat# 10197-1-AP<br>RRID: AB_2118062
Rabbit polyclonal anti-TFCP2	Proteintech	Cat# 15203-1-AP<br>RRID: AB_2199582
Rabbit polyclonal anti-GFP	Proteintech	Cat# 50430-2-AP<br>RRID: AB_11042881
Mouse monoclonal anti-NDRG1	Proteintech	Cat# 26902-1-AP<br>RRID: AB_2880676
Mouse monoclonal anti-EGFR	Proteintech	Cat# 18986-1-AP<br>RRID: AB_10596476
Pan Phospho-Tyrosine Rabbit pAb	ABclonal	Cat# AP0905<br>RRID: AB_2770784
Rabbit polyclonal anti- HA tag	Proteintech	Cat# 51064-2-AP<br>RRID: AB_11042321
Rabbit polyclonal anti- Flag tag	Proteintech	Cat# 20543-1-AP<br>RRID: AB_11232216
Mouse monoclonal anti- Flag tag	Proteintech	Cat# 66008-4-Ig<br>RRID: AB_2918475
Rabbit polyclonal anti-Acetylated Lysine	Cell Signaling Technology	Cat# 9441<br>RRID: AB_331805
HRP-conjugated Goat Anti-Rabbit IgG(H+L)	Proteintech	Cat# SA00001-2<br>RRID: AB_2722564
HRP-conjugated Goat Anti-Mouse IgG(H+L)	Proteintech	Cat# SA00001-1<br>RRID: AB_2722565
VeriBlot IP Detection Reagent (HRP)	Abcam	Cat# ab131366<br>RRID: AB_2892718
Rabbit IgG	Beyotime	Cat# A7016<br>RRID: AB_2905533
Mouse IgG	Beyotime	Cat# A7028<br>RRID: AB_2909433
anti-Acetylated TFCP2-K256	PTM BioLabs	N/A
HDAC2 Rabbit mAb	ABclonal	Cat# A22426<br>RRID: AB_3740224
HDAC3 Rabbit mAb	ABclonal	Cat# A19537

		RRID: AB_2862654
Caveolin-1 Polyclonal antibody	Proteintech	Cat# 16447-1-AP<br>RRID: AB_10732595
LAMP2 Polyclonal antibody	Proteintech	Cat# 27823-1-AP<br>RRID: AB_2880983
**Chemicals**		
Erlotinib	Selleck	S7786
Mocetinostat	Selleck	S1122
Puromycin Dihydrochloride	Beyotime	ST551
Polybrene	Beyotime	C0351
Cycloheximide	Selleck	S7418
Bafilomycin A1	Selleck	S1413
**Bacterial and virus strains**		
DH5a Competent E. coli	Tsingke	Cat#TSC-C14
BL21 (DE3) Competent E. coli	Tsingke	Cat#TSC-E01
**Recombinant DNA**		
Flag-HDAC1-10	Genechem	N/A
Flag-HDAC1(D181A)	Genechem	N/A
Flag-HDAC1(Y72F)	Genechem	N/A
Flag-NDRG1	Genechem	N/A
HA-TFCP2	Genechem	N/A
HA-TFCP2(K256R)	Genechem	N/A

HA-TFCP2(K256Q)	Genechem	N/A
HA-Ub	OBIO	N/A
**Critical commercial assays**		
ChIP Kit Magnetic-One Step	Abcam	ab156907
Dual-Luciferase Reporter Assay System	Promega	E1980
Cell Counting Kit-8	HYCEZMBIO	HYCCK8
**Cell lines**		
HEK293T	Procell	Cat# CL-0005
PANC-1	Procell	Cat#CL-0184
BxPC-3	Procell	Cat#CL-0042
**Software and algorithms**		
ImageJ	ImageJ: Image Processing and	https://imagej.nih.gov/ij/
Graphpad Prism 9.5	Graphpad software	https://www.graphpad.com/

Supplementary Table S3. Clinical information of patients in TMA

Case	Gender	Age	Tumor Location	Tumor Size (cm)	Surgical Procedure	Resection Margin	Pathological Type	Differentiation	LN Examined	LN Positive	T	N	M	Survival Status*	Survival Time (days)
1	Male	63	Head	2.0×1.8×1.5	PD	Negative	PDAC	Moderately	8	0	T1	N0	M0	0	937
2	Male	58	Body/Tail	7.1×6.5×6.0	DP/S	Negative	PDAC	Poorly	21	3	T3	N1	M0	1	582
3	Male	70	Head	2.6×2.4×2.2	PD	Negative	PDAC	Mod-Well	16	0	T2	N0	M0	0	1023
4	Female	66	Head	3.8×3.5×2.8	PD	Negative	PDAC	Moderately	12	1	T2	N1	M0	1	382
5	Male	52	Body/Tail	3.9×3.3×2.6	DP/S	Negative	PDAC	Well	13	0	T2	N0	M0	1	1264
6	Female	74	Head	3.4×3.7×2.2	PD	Negative	PDAC	Poorly	19	3	T2	N1	M0	1	526
7	Female	61	Head	3.8×3.4×2.5	PD	Negative	PDAC	Moderately	8	0	T2	N0	M0	0	923
8	Male	55	Body/Tail	5.4×5.1×4.8	DP/S	Negative	PDAC	Poorly	15	2	T3	N1	M0	1	512
9	Male	68	Head	2.9×2.3×2.1	PD	Negative	PDAC	Well	16	0	T2	N0	M0	0	577
10	Female	49	Head	4.7×3.2×3.0	PD	Negative	PDAC	Poorly	13	1	T3	N1	M0	1	1382
11	Male	72	Head	2.6×2.2×1.5	PD	Negative	PDAC	Poorly	9	0	T2	N0	M0	1	416
12	Female	64	Body/Tail	3.5×3.2×2.5	DP/S	Negative	PDAC	Moderately	16	0	T2	N0	M0	1	955
13	Male	57	Head	2.8×2.6×2.3	PD	Negative	PDAC	Well	12	0	T2	N0	M0	0	1449
14	Female	69	Body/Tail	5.0×4.6×4.2	DP/S	Negative	PDAC	Low-Mod	24	4	T3	N2	M0	1	812
15	Female	60	Body/Tail	4.3×3.9×3.6	DP/S	Negative	PDAC	Moderately	17	0	T3	N0	M0	1	992
16	Male	75	Head	4.6×4.0×3.8	PD	Negative	PDAC	Poorly	13	1	T3	N1	M0	1	693
17	Male	53	Body/Tail	3.5×2.7×2.0	DP/S	Negative	PDAC	Mod-Well	14	0	T2	N0	M0	1	476
18	Female	62	Head	5.7×4.2×3.9	PD	Negative	PDAC	Poorly	22	6	T3	N2	M0	1	915
19	Male	71	Head	4.0×3.7×3.4	PD	Negative	PDAC	Well	9	0	T2	N0	M0	0	659
20	Female	56	Body/Tail	3.0×2.7×2.4	DP/S	Negative	PDAC	Moderately	14	0	T2	N0	M0	0	1265
21	Male	67	Head	5.9×4.5×4.1	PD	Negative	PDAC	Poorly	16	2	T3	N1	M0	1	339
22	Female	59	Body/Tail	3.6×2.3×2.1	DP/S	Negative	PDAC	Mod-Well	15	0	T2	N0	M0	0	1143
23	Male	65	Head	3.4×3.0×2.7	PD	Negative	PDAC	Low-Mod	19	5	T2	N2	M0	1	786
24	Female	48	Head	5.3×4.0×3.8	PD	Negative	PDAC	Poorly	18	3	T3	N1	M0	1	669
25	Male	73	Body/Tail	5.4×4.9×3.6	DP/S	Negative	PDAC	Poorly	14	4	T3	N2	M0	1	642
26	Female	54	Body/Tail	5.5×4.4×4.1	DP/S	Negative	PDAC	Moderately	13	0	T3	N0	M0	0	1326
27	Male	62	Head	3.6×3.1×2.6	PD	Negative	PDAC	Mod-Well	16	0	T2	N0	M0	0	326
28	Female	69	Body/Tail	6.2×5.8×5.5	DP/S	Negative	PDAC	Low-Mod	14	2	T3	N1	M0	1	995
29	Female	58	Body/Tail	3.1×2.9×2.6	DP/S	Negative	PDAC	Moderately	8	0	T2	N0	M0	1	855
30	Female	63	Head	6.9×5.5×5.2	PD	Negative	PDAC	Mod-Well	23	3	T3	N1	M0	1	673
31	Male	70	Head	3.4×3.0×1.3	PD	Negative	PDAC	Well	11	0	T2	N0	M0	0	685
32	Male	52	Head	4.6×4.3×3.0	PD	Negative	PDAC	Well	10	1	T3	N1	M0	1	716
33	Male	74	Body/Tail	5.7×5.1×4.8	DP/S	Negative	PDAC	Well	17	2	T3	N1	M0	1	356
34	Female	61	Body/Tail	3.8×3.5×3	DP/S	Negative	PDAC	Moderately	18	0	T2	N0	M0	1	933
LN: Lymph node			Staging according to AJCC TNM classification					Survival status: 1 = death; 0 = alive							

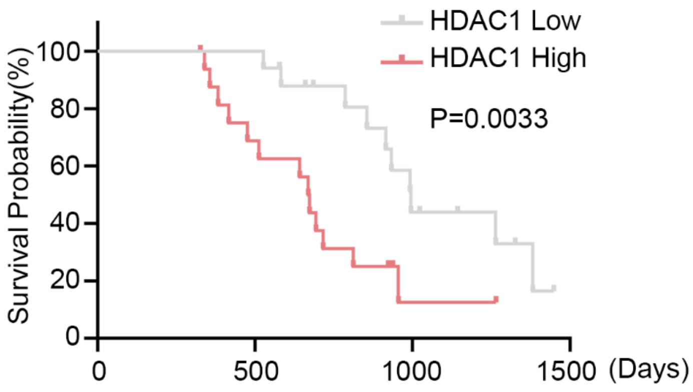

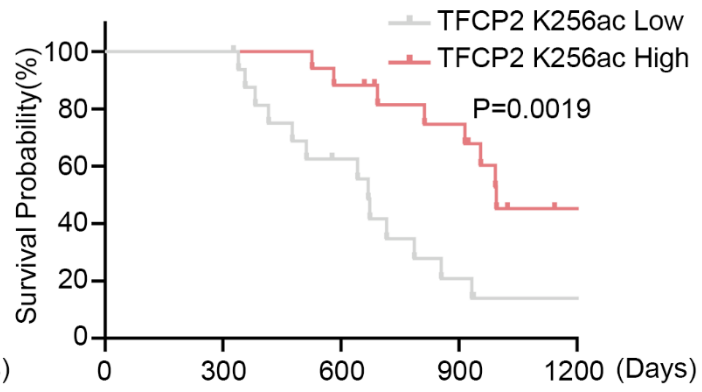

Supplement: Supplementary file 1 — Supplementary figures and tables. [file ijbsv22p4346s1.pdf]
